# Organizing the coactivity structure of the hippocampus from robust to flexible memory

**DOI:** 10.1126/science.adk9611

**Published:** 2024-09-05

**Authors:** Giuseppe P. Gava, Laura Lefèvre, Tabitha Broadbelt, Stephen B. McHugh, Vítor Lopes-dos-Santos, Demi Brizee, Katja Hartwich, Hanna Sjoberg, Pavel V. Perestenko, Robert Toth, Andrew Sharott, David Dupret

**Affiliations:** Medical Research Council Brain Network Dynamics Unit, Nuffield Department of Clinical Neurosciences, University of Oxford, Oxford, United Kingdom

## Abstract

New memories are integrated into prior knowledge of the world. But what if consecutive memories exert opposing demands on the host brain network? We report that acquiring a robust (food-context) memory constrains the hippocampus within a population activity space of highly correlated spike trains that prevents subsequent computation of a flexible (object-location) memory. This densely correlated firing structure developed over repeated mnemonic experience, gradually coupling neurons of the superficial CA1 *pyramidale* sublayer to whole population activity. Applying hippocampal theta-driven closed-loop optogenetic suppression to mitigate this neuronal recruitment during (food-context) memory formation relaxed the topological constraint on hippocampal coactivity and restored subsequent flexible (object-location) memory. These findings uncover an organizational principle for the peer-to-peer coactivity structure of the hippocampal cell population to meet memory demands.

Every day, we use our existing knowledge to guide the actions we make in our environment, integrating new information to gain further knowledge about the world. Therefore, building new memories does not take place in a state of *tabula rasa*, but against a background of prior experiences that have been accumulated across the lifespan and have shaped their host brain networks (1, 2).

The hippocampus network uses the collective activity of the population of its neurons to support everyday memory (3, 4). In principle, the level and structure of the activity coupling between individual neurons could reflect a critical tradeoff between the robustness versus the flexibility of the whole population in processing information. That is, strong peer-to-peer coupling could yield highly correlated spike trains, increasing the consistency of activity patterns within the population for robust memory expression. In contrast, weaker population coupling could release network activity space for new patterns, allowing more diverse mnemonic representations for dynamically adaptable behavior. Owing to convergent innervation on post-synaptic targets (e.g., subiculum, entorhinal cortex), adjusting population coupling to ongoing demand would influence information transmission of hippocampal inputs to downstream reader neurons (3, 5, 6). However, the hippocampus may have to switch between robust versus flexible computations depending on current demands. What are the consequences of placing the hippocampal population into a robust computational mode for subsequent memories that instead require flexible information processing?

To address this question, we first trained six mice (always fed *ad libitum*) to acquire a strong contextual memory. On 16 consecutive days, mice explored two arenas (Fig. 1A, B). During the first 10 days (‘Food-context conditioning’), we paired one arena (context *X*) with two regular chow pellets (fig. S1A). The other arena (context *Y*) contained one chow pellet and one high-fat-diet (Hfd) pellet (fig. S1A), which mice encountered for the first time and did not eat much on day 1 (Fig. 1C). By repeating these foraging sessions on each subsequent conditioning day, mice showed escalated food intake in context *Y* (Fig. 1C; from day 1 to day 10, a fold-change of 21.62 ± 6.70 versus 0.63 ± 0.24 in context *Y* versus *X*; mean ± s.e.m.). Food locations were randomized every day in each context to promote mnemonic association of food items not to fixed places but whole contexts (fig. S1B). To probe discriminative food-context association, we then measured the propensity of these mice to express context-biased feeding. By providing both arenas with new food items in post-conditioning days (‘Novel food test’; days 11 and 12; Fig. 1B and fig. S1C), we observed higher novel food intake in context *Y* compared to context *X* (Fig. 1D; a fold-change of 3.47 ± 1.40 in context *Y* versus *X*; mean ± s.e.m.). Thus, mice in the Hfd-conditioned context readily overcame the rodent natural tendency to express food neophobia (7, 8). Mice also exhibited lower Hfd intake when provided in a third arena (context *Z*) never paired with any food (fig. S1D). Body weight remained stable across days (fig. S1E).

We next switched task demand to assess novelty detection in these contexts paired with food. For this, we used a hippocampus-dependent continuous Novel Object Recognition task (‘cNOR test’; from day 13 to day 16; Fig. 1A, B and fig. S2A-C). On each cNOR day, we re-exposed mice to either context *X* or *Y* (‘Re-exposure’; without food) before they encountered four novel objects (‘Sampling’; Fig. 1A, B and fig. S2A, B). Across four more exploration sessions that day, we iteratively replaced one of the initially sampled objects with a new one (‘Testing’). This procedure yielded a set of three familiar (already-seen) objects and one novel (first time-seen) object in each cNOR test (Fig. 1B and fig. S2A). We measured novelty detection in each cNOR test *n* using the time spent exploring the novel object over the total time spent on all four objects, thereby probing memory for objects explored in session *n* − 1. Mice showed novel object preference in context *X* but not in context *Y* (Fig. 1E). Exploratory behavior measured by locomotor speed, distance travelled, and time spent with objects did not differ across contexts (fig. S2D-H). Altogether, the first demand in this 2-memory paradigm was for animals to repeatedly associate food resources to selective contexts across many days, yielding a robust memory able to shape contextual feeding. This later prevented mice from coping with a second demand: to dynamically update another memory for continually detecting novel items across many sessions each day. Thus, the robust (food) memory acquired in context *Y* interfered with the subsequent flexible (object-location) memory in that context.

## Robust memory increases neuronal coactivity and population coupling in the hippocampus

**Fig. 1:**
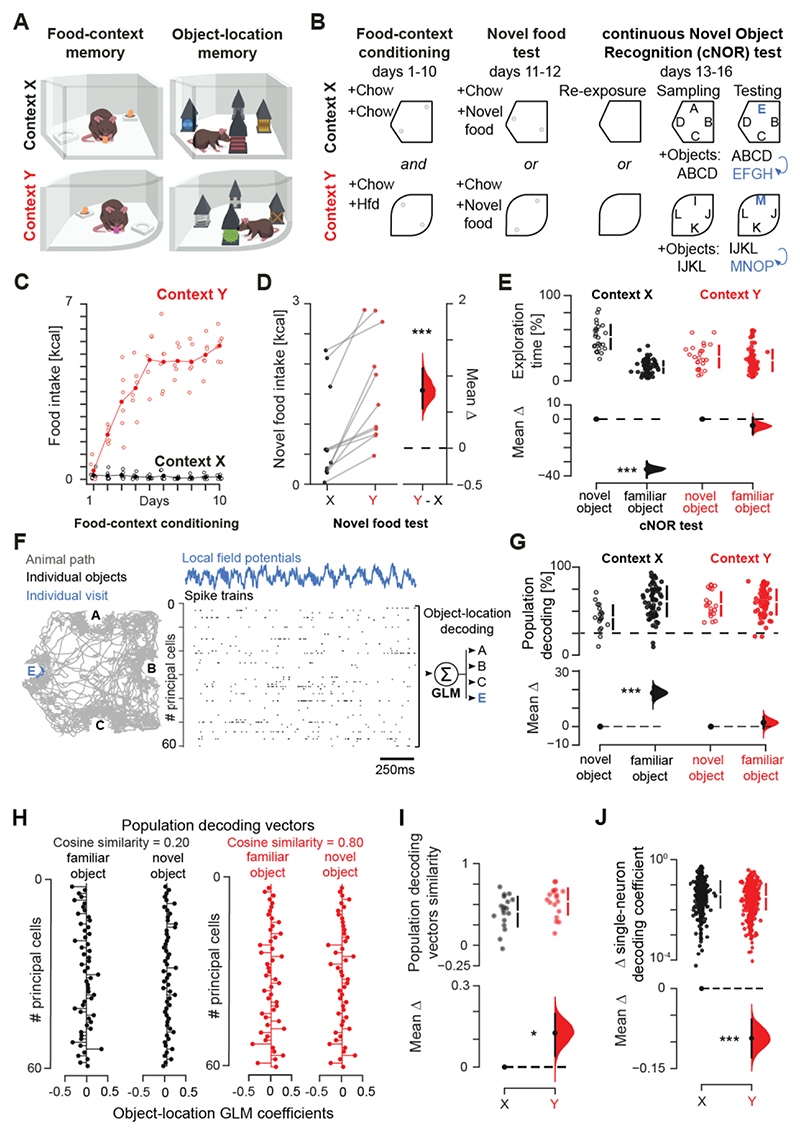
Robust contextual (food) memory prevents subsequent flexible (object) memory. **(A, B)** Behavioral tasks with open-field contexts **(A)** and multiday layout **(B)** for a two-memory paradigm. **(C)** Animals’ food intake during contextual conditioning (each data point represents one mouse). (**D)** Estimation plot showing the effect size for the difference in novel food intake between context *X* and *Y* after conditioning. (**E**) Percentage of time exploring novel versus familiar objects during cNOR tests in context *X* or *Y*. (**F**) Schematic of the GLM predicting the identity of each object-location compound from population vectors of theta-nested principal cell spiking. A sample of CA1 ensemble spike data for one object visit (blue trace) is shown. (**G**) Estimation plot showing the classification accuracy of object-location compound in test *n* by GLM trained in session *n* − 1 (each data point represents one object). **(H)** Example pairs of population decoding vectors containing the neuron-wise GLM coefficients for the familiar versus a novel object at the same location in context *X* versus *Y* (each data point represents one neuron). **(I, J)** Estimation plots showing the cosine similarity between familiar and novel object-location GLM vectors **(I)** and the change (update) in individual neuron contribution to population object-location decoding **(J)** across two consecutive cNOR tests in either context. For each estimation plot: *Upper* (*Left* for D), raw data (*points*) with mean±SD (*vertical lines*); *Lower* (*Right* for D), mean difference (*black-dot*) with 95% CI (*black-ticks*) and bootstrapped sampling-error distribution (*filled-curve*) with respect to the (left-most) group-reference (*horizontal dashed line*; see Methods). ***P<0.001, *P<0.05, two-sided paired permutation test. N = 6 mice, 2506 CA1 principal cells.

We aimed to identify the neuronal correlates of this cross-memory interference. During active behavior, groups of principal cells recruited from the population of hippocampal neurons cooperate within the timeframe of theta-band (5–12Hz) oscillations to support codes and computations for memory (3). We recorded cell ensembles and local field potentials in the CA1 *stratum pyramidale* of these mice. Using the action potentials discharged by principal cells in theta cycles during exploration of each object in cNOR days (Fig. 1F and fig. S2I), a generalized linear model (GLM) trained on session *n* − 1 and applied in test *n* identified each object-location compound with up to 93.5% accuracy (range 9.4 – 93.5 %; mean 55.0 % compared to a chance level of 25.0 %; mean ± s.e.m. number of principal cells = 51.1 ± 3.7 per GLM), consistent with work showing population-level object representation in the hippocampus (9, 10). In context *X*, the mean accuracy of this population-level decoding started at 38.6 ± 3.2 % for the novel object-location compound (Fig. 1G) and improved by the following trial (fig. S2J), indicating rapid mnemonic integration of each object. This across-test gain in object-location representation did not occur in context *Y* (Fig. 1G and fig. S2J). In fact, the mean decoding accuracy there started at higher levels for the novel object-location (54.9 ± 3.4 %; P<0.001, two-sided paired permutation test, compared to context *X*), without significant changes in the following tests (Fig. 1G and fig. S2J). In both context *X* and *Y*, object-location memory was not affected by provision of Hfd (in neutral context *Z*) beforehand that day (fig. S3). Compared to context *X*, the contribution of individual cells to each novel object-location decoding during test *n* in context *Y* resembled its previous one expressed in session *n* − 1 at that location while having another (familiar) object. This was reported by the higher similarity between the population decoding vector that contained the set of neuron-wise GLM coefficients representing the novel object during test *n* in context *Y* versus that representing the familiar object at the same location during session *n* − 1 (Fig. 1H, I). In line with this observation, the across-test modulation in single-neuron contributions to population decoding vectors when encountering a novel object (i.e., the changes in the magnitude of individual GLM coefficients) was weaker in context *Y* compared to *X* (Fig. 1J).

**Fig. 2:**
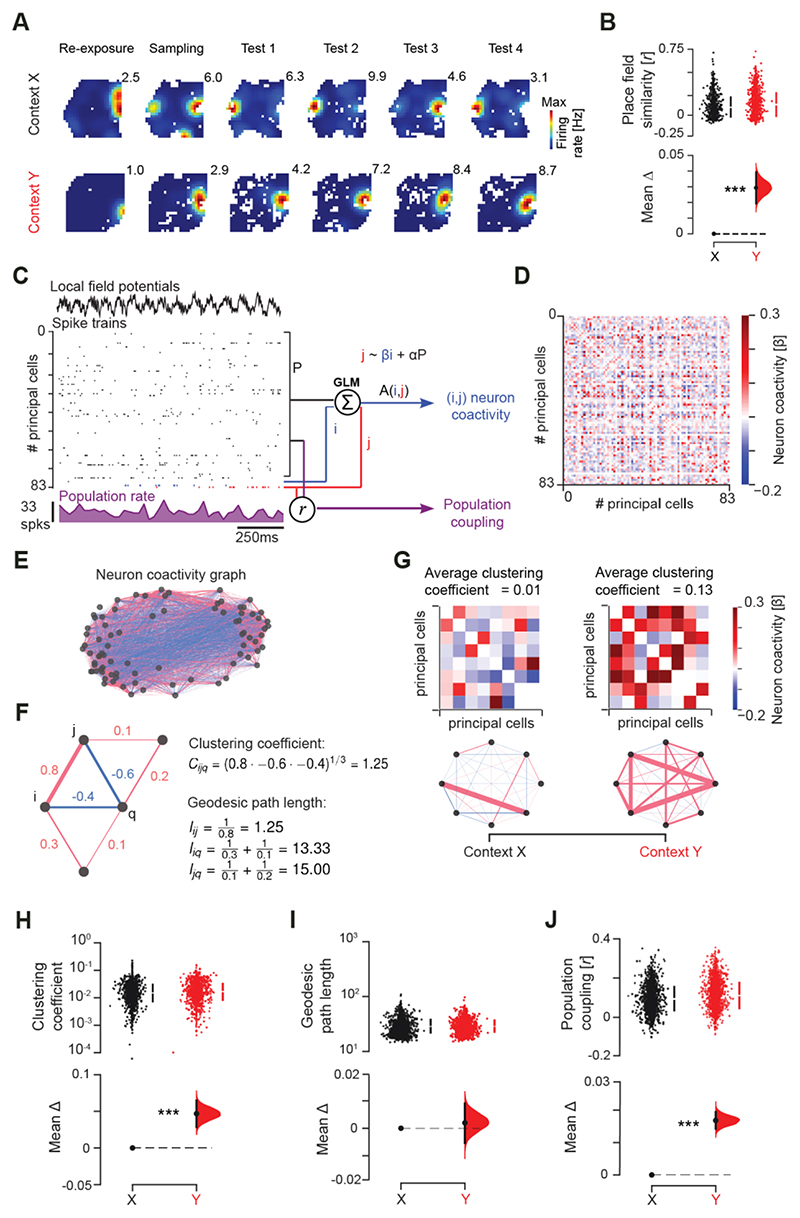
Robust memory increases neuronal coactivity and population coupling. **(A)** Example firing maps across the consecutive cNOR sessions for one mouse day in context *X* (top) versus context *Y* (bottom). Each row shows one principal cell (numbers indicate peak rate for each map). **(B)** Estimation plot showing the place field similarity for the pairs of place maps expressed by individual cells across two contiguous cNOR sessions (e.g., Sampling and Test 1) in context *X* versus context *Y* (each data point represents one cell). (**C**) Schematic of the population-level analyses (see methods). Coactivity between any two (*i, j*) neurons measured as the *β* regression weight from the GLM assessing their firing relationship while accounting for network-level modulation using the sum of the remaining cells in the population (to estimate neuron pair (*i, j*) coactivity beyond the population rate). Population coupling of each cell measured as the Pearson correlation coefficient between its theta-binned spike train and the cumulative activity of the remaining cells. **(D-G)** Example adjacency matrix of *β* regression weights (D) and corresponding coactivity graph (E) using the procedure depicted in (C) to access the neuron (*i, j*) pairwise coactivity structure of the population in each context. Example subset of a coactivity graph (F) with five neurons (nodes) and their pairwise coactivity values (edges with numbers); the clustering coefficient *C*_*ijq*_ of neuron *i* forming one example triad with neurons *j* and *q* is calculated along with the geodesic path lengths *l*_*ij*_, *l*_*iq*_, *l*_*jq*_. Shown in (G) is a subset of adjacency matrices representing contexts *X* and *Y* (top), along with their average clustering coefficients and motifs of coactivity (bottom). (**H-J)** Estimation plots showing that the population coactivity structure is tighter in context *Y* than *X*, as reported by the higher clustering coefficient of neuronal graphs containing stronger triads of coactive neurons (H), without a significant change in geodesic path length (I), along with stronger population coupling (J). For each estimation plot: *Upper*, raw data (*points*) with mean±SD (*vertical lines*); *Lower*, mean difference (*black-dot*) with 95% CI (*black-ticks*) and bootstrapped sampling-error distribution (*filled-curve*) with respect to the (left-most) group-reference (*horizontal dashed line*; see Methods). ***P<0.001, two-sided paired permutation test. N = 6 mice, 2506 CA1 principal cells.

We next computed the CA1 place maps expressed during the cNOR task (Fig. 2A and fig. S4A). Context *Y* did not exhibit an over-representation of the randomized Hfd location (fig. S4B). By quantifying the cross-session similarity of place maps from re-exposure to sampling to individual object recognition tests on each day, we observed that CA1 principal cells exhibited higher place map stability (i.e., lower spatial remapping) across contiguous sessions in context *Y* (Fig. 2B and fig. S4C). With the GLM object-location decoding, this result on the hippocampal place code supported the notion of a more rigid memory in the Hfd-paired context *Y*, but a flexible (cross-session updated) memory in context *X*.

We hypothesized that this representational rigidity reflects the organization of the population activity into a non-permissive structure. An operational principle serving many brain functions, including memory, is to leverage the collective activity of neural populations as an emergent property beyond that of individual cells (11–14). We reasoned that Hfd context-conditioning yielded highly correlated firing patterns that created a dense network activity space for strong contextual (food) memory. But this later conflicted with the switch to a different demand where continually processing familiar versus novel stimuli would instead require sparser, weakly correlated patterns for disentangling discrete (object-location) representations. The correlational structure of the population activity was markedly different in context *Y* compared to *X* (Fig. 2C-J). We quantified the coactivity association of each cell pair (*i, j*) by predicting the theta-nested spike discharge of neuron *j* from the activity of neuron *i* while regressing out the activity of the remaining population (Fig. 2C). This procedure returned a matrix of *β* regression weights (Fig. 2D) that represented the neurons pairwise coactivity structure of the population in each context. For both context *X* and *Y* we constructed weighted neuronal graphs (with no self-connections) where each node is a cell and the edge linking any two nodes represents the coactivity of that cell pair (Fig. 2D-G). Neuronal graphs contained stronger triads of coactive nodes in context *Y* than *X*, as reported by higher clustering coefficients (Fig. 2H and fig. S5A; mean increase (95% CI): 11.0% (7.3–14.1%)). The population coactivity strength level, calculated for each node as the average weight of all its edges, was higher in *Y* (fig. S5B), with no difference in the mean neurons’ firing rate across contexts (fig. S5C). The hippocampal population exhibited this denser coactivity structure in context *Y* without a reduction in geodesic path length, calculated as the mean shortest path between any two nodes (Fig. 2I and fig. S5A) (15). This suggested that the hippocampus, which usually displays the features of a small-world network (fig. S6A-C) that could allow for flexible information updating through efficient synchronization, has in fact acquired in context *Y* the rigidity of a more coherent, lattice-like network (fig. S6D-F) (16–18). These neuronal graphs that are composed of both correlated and anti-correlated spike trains (i.e., positive and negative edges) indeed showed more stable population activity patterns in context *Y*, as suggested by higher structural balance (fig. S6G-I). These topological alterations developed across the 10 conditioning days (fig. S7A) to continue altering the level and structure of population coactivity in post-conditioning days (fig. S7B), affecting the baseline re-exposure to context *Y* prior to any testing. Hippocampal graphs yet shared some common correlation structure across contexts (fig. S8), suggesting a coactivity backbone for cross-context generalization.

To explore the development of a dense population activity structure in context *Y*, we investigated the one-to-many relationship between individual neurons and the rest of the population. We measured the coupling of each principal cell instantaneous firing rate in theta cycles to the concomitant summed activity of all other recorded cell members of the population (‘population rate;’ Fig. 2C). Consistent with the higher topological clustering (Fig. 2H), the average population coupling of individual neurons was stronger in context *Y* (Fig. 2J and fig. S5A; mean increase (95% CI): 16.6% (12.6–20.8%)). This increased population coupling reflected a stronger cross-neuron spiking relationship: shuffling the spike times across neurons and theta cycles, while preserving each neuron’s mean rate and the population rate distribution, cancelled the increased population coupling seen in context *Y* (fig. S9A). This heightened coupling developed across the 10 conditioning days to mark the re-exposure to context *Y* during post-conditioning days even before any test (fig. S9B). Restricting food-context conditioning to two days in five additional mice did not alter subsequent cNOR memory (fig. S10A-D), allowing successful object-location decoding in both contexts with similar CA1 population activity level and structure (fig. S10E–J). Contextual food conditioning thus seemed to increase the recruitment of principal cells as “choristers of a larger hippocampal orchestra” (19) when daily repeated for well over a week.

## Mitigating neuronal recruitment during robust memory formation relaxes hippocampal coactivity and restores flexible memory

**Fig. 3:**
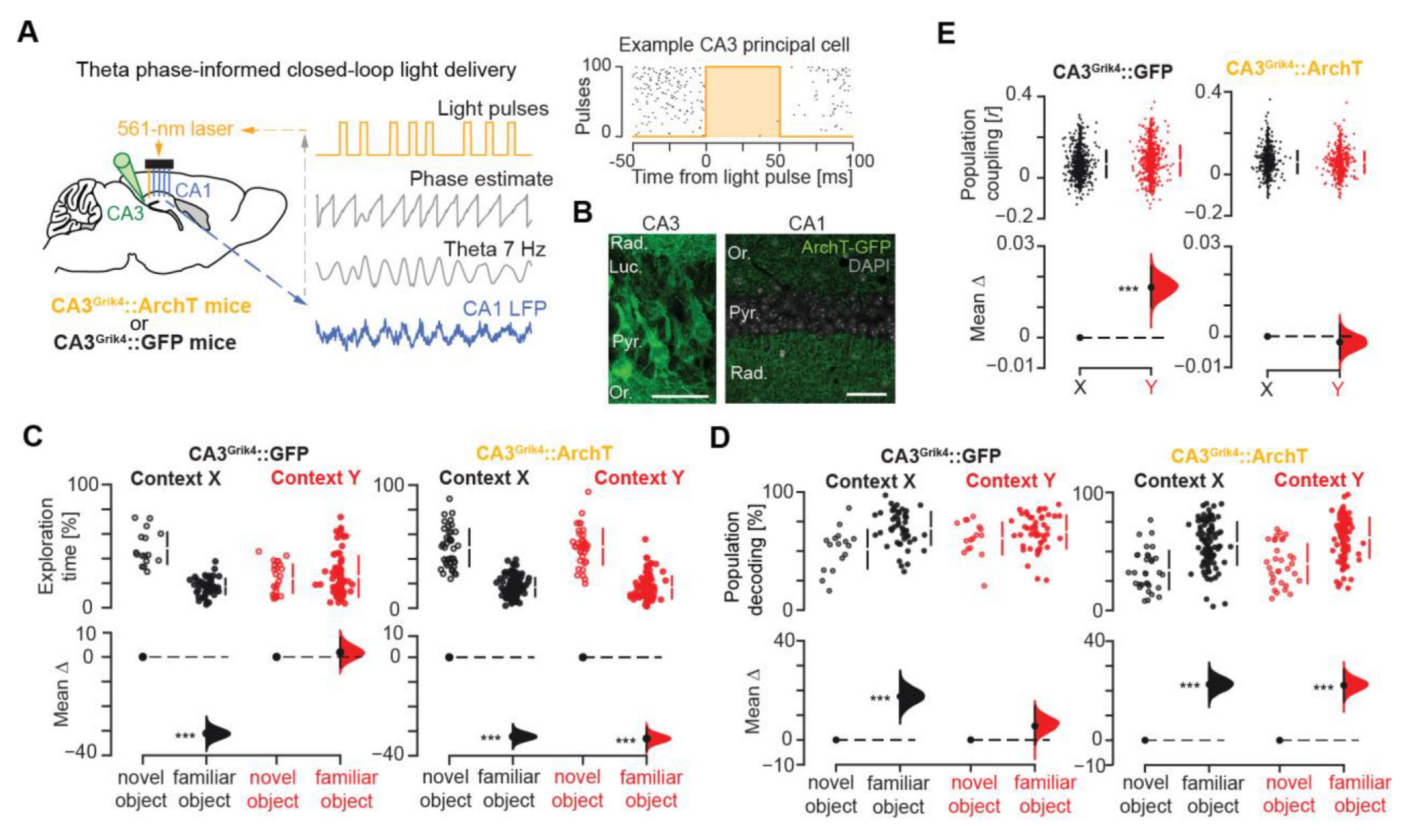
CA1 population coupling requires CA3 during food-context conditioning. (**A, B**) Optogenetic targeting of CA3 with either ArchT-GFP (in CA3^Grik4^::ArchT mice) or GFP-only (in CA3^Grik4^::GFP mice) combined with theta phase-informed light delivery for closed-loop suppression of CA3 during Hfd-context *Y* conditioning (A). CA3 principal cells transduced with ArchT-GFP (B, *left*) and projecting to CA1 (B, *right*; Stratum *oriens*, Or.; *pyramidale*, Pyr.; *radiatum*, Rad., *lucidum*, Luc.; cell nuclei stained with DAPI; scale bar, 50 μm). (**C-E**) Estimation plots showing that in CA3^Grik4^::ArchT mice, but not in control CA3^Grik4^::GFP mice, applying this optogenetic intervention throughout the 10-day Hfd conditioning subsequently restored in context *Y* the behavioral cNOR performance (C) with CA1 population object-location decoding (D) and activity coupling (E) to levels seen in context *X*. For each estimation plot: *Upper*, raw data (*points*) with mean±SD (*vertical lines*); *Lower*, mean difference (*black-dot*) with 95% CI (*black-ticks*) and bootstrapped sampling-error distribution (*filled-curve*) with respect to the (left-most) group-reference (*horizontal dashed line*; see Methods). ***P<0.001, two-sided paired permutation test. N = 1548 CA1 neurons from 4 CA3^Grik4^::ArchT mice versus 881 CA1 neurons from 2 CA3^Grik4^::GFP mice.

We sought to relate hippocampal population activity to memory expression more directly by first manipulating an underlying neural pathway. The CA3 region features extensive excitatory recurrent connections and Hebbian synaptic plasticity (20–23). It could therefore promote population coupling in the downstream CA1, which has little to no recurrent excitation (6, 24). To test this, we transduced CA3 principal cells with the neural silencer Archaerhodopsin-T in four Grik4-Cre mice versus the GFP-only control in two Grik4-Cre mice (Fig. 3A, B). Bilateral implantation of tetrodes combined with optic fibers allowed monitoring of CA1 ensembles while actuating a theta phase-informed controller for real-time suppression of CA3 principal cells (Fig. 3A and fig. S11A-C). In CA3^Grik4^::ArchT mice, but not CA3^Grik4^::GFP mice, applying this closed-loop intervention during each Hfd conditioning session subsequently restored in post-conditioning test days object-location memory with CA1 place map similarity (cross cNOR-session remapping) and population coupling in context *Y* to levels seen in context *X* (Fig. 3C-E and fig. S11D-H).

**Fig. 4:**
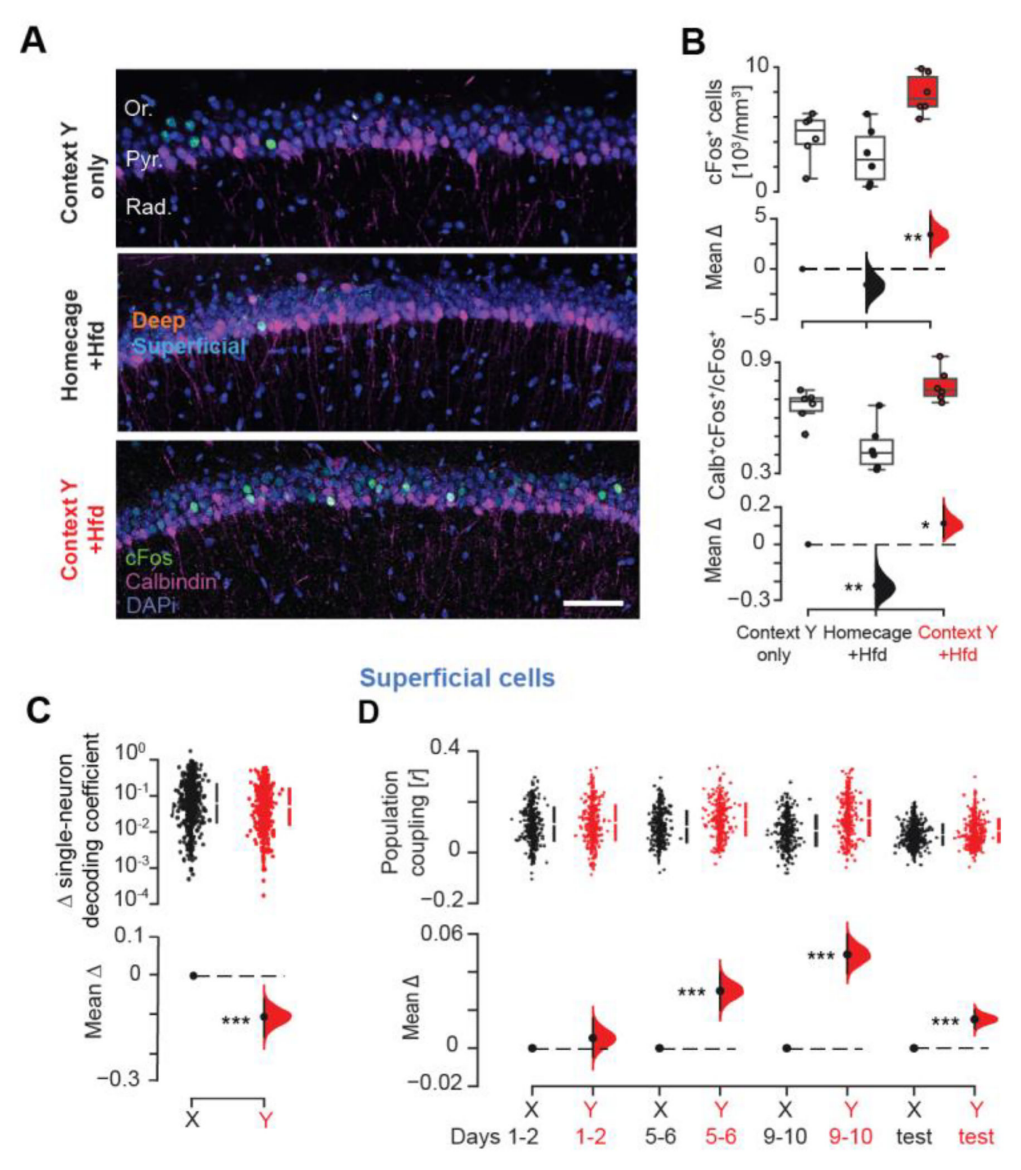
Contextual food memory recruits CA1 superficial *pyramidale* sublayer cells. (**A, B**) cFos–expressing CA1 neurons with Calbindin-1 delineated superficial *pyramidale* sublayer for three mice after 10-day exposure to either context *Y* only, homecage with Hfd, or context *Y* with Hfd (A; stratum *oriens*, Or.; *pyramidale*, Pyr.; *radiatum*, Rad.; cell nuclei stained with DAPI; scale bar, 50 μm; see also fig. S12), and quantification of cFos^+^ cell density in CA1 *pyramidale* (B; *top*; each datum represents one mouse; N = 18 mice, 6 mice per condition) with corresponding proportion of cFos^+^ Calbindin^+^ cells in the CA1 superficial *pyramidale* sublayer (B; *bottom*). (**C, D**) Estimation plots showing that CA1 superficial *pyramidale* sublayer cells have reduced cross-test change (update) in their contribution to object-location decoding (C) and increased population coupling (D) in context *Y* compared to context *X* (N = 6 mice, 1871 CA1 superficial cells). ***P<0.001, **P<0.01, *P<0.05, two-sided paired permutation test.

We then examined the CA1 population after contextual food conditioning. Using the activity-dependent immediate-early-gene cFos, we quantified neuron recruitment in the CA1 *pyramidale* layer of six mice exposed to context *Y* with Hfd for 10 days (Fig. 4A and fig. S12). In parallel, six control mice explored context *Y* without food while six others ate Hfd in their homecage. Mice undergoing Hfd-context *Y* conditioning showed higher density of cFos^+^ neurons compared to both controls (Fig. 4B). Contrasting cFos expression in the superficial versus the deep *pyramidale* sublayers using the marker Calbindin 1 (Fig. 4A) (25–27) suggested that Hfd-context conditioning preferentially recruited CA1 superficial cells (Fig. 4B). This is consistent with recent studies highlighting that CA1 pyramidal cells segregate along the anatomical axes of the hippocampus (e.g., with respect to molecular markers, neural connectivity, and electrophysiological properties), indicating a functional specialization based on somatic location (25–35).

We identified neurons recorded in the CA1 superficial *pyramidale* sublayer, using the electrophysiological profile of each tetrode (fig. S13) (15). The representational rigidity affecting object-location memory update in context *Y* (Fig. 1J) corresponded to reduced modulation in the individual contribution of superficial cells to the population decoding from cNOR session *n* − 1 to test *n* (Fig. 4C and fig. S14A). This suggested that superficial cell population did not distinguish the novel object from the familiar previously encountered at the same location in context *Y*, only representing the location itself. In line with this, the spatial map rigidity (i.e., higher place field stability) observed across cNOR sessions in the Hfd-conditioned context *Y* (Fig. 2B) was explained by superficial cell maps (fig. S14B), with no representational bias to the Hfd location (fig. S14C). Superficial cells formed stronger coactivity triads in the network (fig. S15A-E), increasing their population coupling in context *Y* (Fig. 4D and fig. S15F-H). Deep *pyramidale* sublayer cells did not show such enhanced activity profile (fig. S15). The CA1 representational rigidity that prevented flexible object-location memory in context *Y* following Hfd conditioning was thus primarily explained by enhanced recruitment of superficial *pyramidale* sublayer cells.

**Fig. 5:**
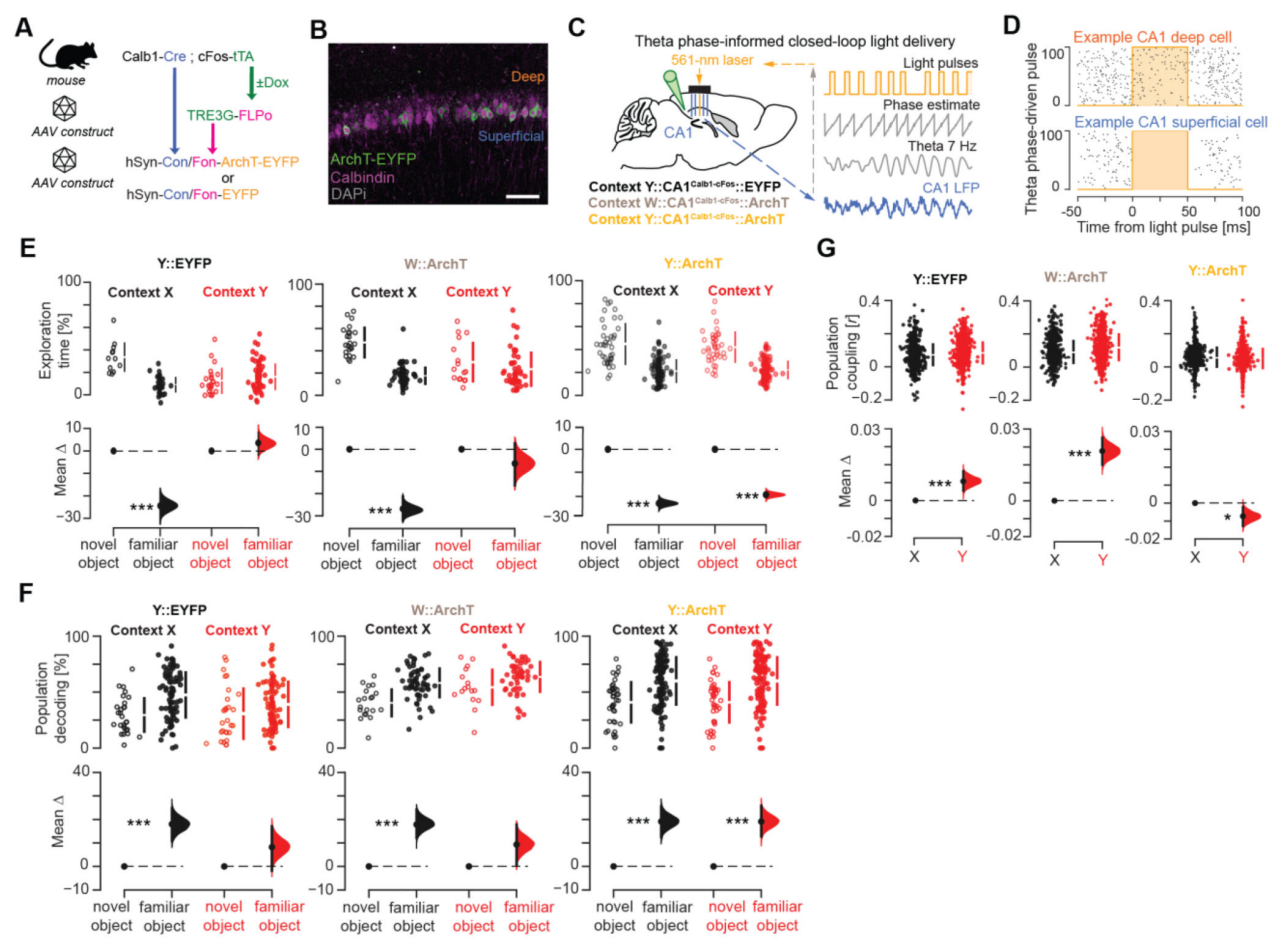
Adjusting hippocampal population coactivity restores flexible memory. **(A, B)** Intersectional optogenetic strategy (A) for activity-dependent tagging of CA1 Calb1 neurons with either ArchT-EYFP or EYFP-only during Hfd conditioning in context *Y*, or with ArchT-EYFP during exploration of neutral context *W* (B; *green*, ArchT-EYFP; *magenta*, Calbindin-1; *gray*, DAPI; see also fig. S17). **(C, D)** Closed-loop CA1 light delivery controller (C) combined with the optogenetic strategy shown in (A) to suppress superficial *pyramidale* sublayer cells at their preferred theta phase (fig. S18) during Hfd-context *Y* conditioning. (D) shows example spiking activity for a deep (top) versus a superficial (bottom) *pyramidale* sublayer cell with respect to theta phase-driven light delivery. **(E-G)** Estimation plots. For the context *Y*-tagged optogenetically adjusted ArchT mice, but not for the context *Y*-tagged EYFP-only mice nor the context *W*-tagged ArchT mice, this cell type-selective, network pattern-informed intervention restored cNOR performance (E) along with population object-location decoding (F) and population coupling (G) in context *Y*. ***P<0.001, *P<0.05, two-sided paired permutation test. N = 1097 CA1 principal cells from 5 Context*Y*::CA1^Calb1-cFos^::ArchT mice (*Y*::ArchT) versus 1007 from 4 Context*Y*::CA1^Calb1-cFos^::EYFP mice (*Y*::EYFP) and 683 from 3 Context*W*::CA1^Calb1-cFos^::ArchT mice (*W*::ArchT).

We hypothesized that harnessing the rise in population coupling during robust contextual conditioning would restore subsequent flexible memory. We used an intersectional optogenetic strategy to target cells recruited in the CA1 superficial *pyramidale* sublayer during Hfd-context *Y* conditioning. CA1 superficial *pyramidale* sublayer cells are genetically defined by the molecular marker Calbindin-1 (25, 27, 34). They express cFos during contextual Hfd conditioning (Fig. 4A, B). We thus bred double-transgenic Calb1-Cre;cFos-tTA mice and generated a viral construct for the two-term Boolean logic (36) expression of the yellow light-driven neural silencer ArchT-EYFP (or its EYFP-only control) dependent on the two recombinases Cre and FlpO (Fig. 5A).

We transduced the CA1 of these mice with this construct together with a second construct allowing the tTA-dependent expression of FlpO (Fig. 5A) for lasting optogenetic tagging of CA1 superficial *pyramidale* sublayer cells with either ArchT-EYFP (in five Context*Y*::CA1^Calb1-cFos^::ArchT mice) or the control EYFP-only (in four Context*Y*::CA1^Calb1-cFos^::EYFP mice) from the onset of Hfd conditioning in context *Y* (Fig. 5B and figs. S16, S17). We also tagged with ArchT-EYFP the set of CA1 superficial cells associated with a task-unrelated arena (context *W*) in another group of control mice (three Context*W*::CA1^Calb1-cFos^::ArchT mice; fig. S17). CA1 superficial principal cells, which preferentially fire action potentials at the trough of theta cycles (fig. S18A-C) (15, 33, 35), exhibited an increased theta modulation across conditioning days (fig. S18D). We thus combined this intersectional strategy with our theta phase-informed controller for closed-loop silencing of CA1 superficial cells during Hfd conditioning sessions (Fig. 5C,D and fig. S18E,F). Despite undergoing 10-day Hfd feeding in context *Y* (fig. S19A), this cell type-defined, network pattern-informed intervention subsequently restored in Context*Y*::CA1^Calb1-cFos^::ArchT mice natural behavioral response to novel food (fig. S19B) and object-location memory (Fig. 5E). This was not the case in Context*Y*::CA1^Calb1-cFos^::EYFP mice and Context*W*::CA1^Calb1-cFos^::ArchT mice that showed impaired object-location memory in context *Y* (Fig. 5E). In line with this behavioral outcome, Context*Y*::CA1^Calb1-cFos^::ArchT mice recovered functional object-location decoding and population coupling (Fig. 5F,G and figs. S19C-E, S20).

## Discussion

Our results show that a robust (food-context) memory raises the population coupling of CA1 superficial *pyramidale* sublayer neurons, creating a dense network coactivity structure. CA1 pyramidal cells, the primary output of the hippocampus, segregate along the anatomical axes with different activity behaviors, indicating that cells arranged along the radial axis contribute differentially to information processing (25–31, 35). Compared to the deep, the superficial sublayer is enriched in context-modulated cells with slower response dynamics to environmental changes (28, 29), being more active in cue-poor environments and preferentially using a rate code driven by intra-hippocampal inputs; while deep cells are more active in cue-rich environments and use a phase code driven by entorhinal inputs (30). Our findings show that the hippocampus network preferentially engages superficial cells in strong peer-to-peer coactivity for robust contextual memory, at the risk of subsequent mnemonic rigidity. This finding is consistent with recent studies showing that CA1 superficial cells are more recruited into replay events and show stronger synaptic potentiation after novel experiences compared to deep cells (37, 38). The dense coactivity structure emerged over many conditioning days and could prevent the network to switch between alternative coding schemes or cell assemblies, and override representations by the deep sublayer counterparts (28, 30). The development of this coactivity structure required upstream CA3 activity, consistent with the observation that Schaffer collateral excitation is stronger in calbindin-expressing CA1 principal cells (25). With respect to subsequent behavior, this acquired hippocampal topology of heightened coactivity related to strong contextual (feeding) response and impaired novel information processing. Neural networks have been proposed to fall into the broad class of ‘small-world’ networks, a middle ground between regular and random networks where the combination of high clustering of elements (a property of regular networks) with short path lengths between elements (a property of random networks) would allow important properties of complex networks such as increased computational power and effective synchronizability (16, 17, 39–41). Our observation that repeated food-context conditioning affects the coactivity structure of the network by increasing neuronal clustering without shortening node-to-node paths suggests that the hippocampal topology can deviate from a small-world toward a more coherent, regular lattice. This way, the joint activity of an increased number of neurons operating as a cohesive population would permit robust information flow to downstream receiver neurons, possibly at the expense of other (e.g., novel) input channels.

The hippocampus could broadcast this heightened coactivity to several recipient circuits and reader neurons. For instance, recent work points to a contribution of the nucleus accumbens in translating hippocampal dynamics of appetitive memory into a behavioral readout (42) or the hypothalamus in driving non-homeostatic contextual feeding (43). The clustered spiking activity that developed in the hippocampus across food-context conditioning days is also likely to influence neocortical circuits for memory storage via systems consolidation (44, 45). With hippocampal support neuronal ensembles in prefrontal cortex can be rapidly formed to then undergo a process of functional maturation over weeks (46). This maturation could allow prefrontal cortex ensembles to converge onto a lower-dimensional activity space to extract latent rules and common relational features across multiple experiences, gradually developing a knowledge structure of the world (47, 48). Importantly, the instantiation of this highly clustered topology can be prevented: applying cell type-selective, network pattern-informed neuronal suppression during contextual learning rebalances population activity and restores flexible memory. Together, these findings suggest that the plastic organization of hippocampal coactivity supports a network tradeoff between robust and flexible computations, shaping continual integration of new memories and their adaptability to cognitive demands.

## Supplementary Material

Supplementary Material

## Data Availability

All data are available in the manuscript or the supplementary materials. The c-fos-tTA mouse line was generated at The Scripps Research Institute and obtained from Dr. L.G. Reijmers at Tufts University under a material transfer agreement with The Scripps Research Institute. Hippocampal recordings (49) and Jupyter notebooks for ensemble analysis (50), and 3D printable objects for cNOR task (51) are available via the Medical Research Council (MRC) Brain Network Dynamics Unit (BNDU) data sharing platform (https://data.mrc.ox.ac.uk/mrcbndu/data-sets/search).

## One-Sentence Summary

The density of peer-to-peer neuronal coactivity determines robust versus flexible memory expression in the hippocampus.

## References

[1] Schacter DL, Addis DR, Buckner RL (2007). Nat Rev Neurosci.

[2] Brod G, Werkle-Bergner M, Shing YL (2013). Frontiers in Behavioral Neuroscience.

[3] Buzsáki G (2010). Neuron.

[4] Mau W, Hasselmo ME, Cai DJ (2020). eLife.

[5] Barnes CA, McNaughton BL, Mizumori SJY, Leonard BW, Lin L-H Progress in Brain Research.

[6] van Strien NM, Cappaert NLM, Witter MP (2009). Nat Rev Neurosci.

[7] Kronenberger J-P, Médioni J (1985). Behavioural Processes.

[8] Barnett SA (1958). British Journal of Psychology.

[9] Nagelhus A, Andersson SO, Cogno SG, Moser EI, Moser M-B (2023). Neuron.

[10] Vandrey B, Duncan S, Ainge JA (2021). Hippocampus.

[11] El-Gaby M (2021). Nat Neurosci.

[12] van de Ven GM, Trouche S, McNamara CG, Allen K, Dupret D (2016). Neuron.

[13] Liu C, Todorova R, Tang W, Oliva A, Fernandez-Ruiz A (2023). Science.

[14] Panzeri S, Moroni M, Safaai H, Harvey CD (2022). Nat Rev Neurosci.

[15] Gava GP (2021). Nature Neuroscience.

[16] Watts DJ, Strogatz SH (1998). Nature.

[17] Newman MEJ (2003). SIAM Rev.

[18] Muldoon SF, Bridgeford EW, Bassett DS (2016). Scientific Reports.

[19] Okun M (2015). Nature.

[20] Wittner L, Henze DA, Záborszky L, Buzsáki G (2007). Brain Struct Funct.

[21] Ishizuka N, Weber J, Amaral DG (1990). J Comp Neurol.

[22] Bains JS, Longacher JM, Staley KJ (1999). Nat Neurosci.

[23] Zalutsky RA, Nicoll RA (1990). Science.

[24] Amaral D, Lavenex P, Andersen P, Morris R, Amaral D, Bliss T, O’Keefe J (2006). The Hippocampus Book.

[25] Soltesz I, Losonczy A (2018). Nat Neurosci.

[26] Cembrowski MS, Spruston N (2019). Nature Reviews Neuroscience.

[27] Valero M (2015). Nature Neuroscience.

[28] Danielson NB (2016). Neuron.

[29] Geiller T, Fattahi M, Choi J-S, Royer S (2017). Nat Commun.

[30] Sharif F, Tayebi B, Buzsáki G, Royer S, Fernandez-Ruiz A (2021). Neuron.

[31] Masurkar AV (2017). Cell Reports.

[32] Masurkar AV (2020). Journal of Neurophysiology.

[33] Grosmark AD, Buzsáki G (2016). Science.

[34] Cembrowski MS (2016). Neuron.

[35] Navas-Olive A (2020). Nature Communications.

[36] Fenno LE (2014). Nature Methods.

[37] Berndt M, Trusel M, Roberts TF, Pfeiffer BE, Volk LJ (2023). Neuron.

[38] Harvey RE, Robinson HL, Liu C, Oliva A, Fernandez-Ruiz A (2023). Neuron.

[39] Bullmore E, Sporns O (2009). Nat Rev Neurosci.

[40] Bassett DS, Sporns O (2017). Nature Neuroscience.

[41] Barahona M, Pecora LM (2002). Phys Rev Lett.

[42] Trouche S (2019). Cell.

[43] Mohammad H (2021). Nat Neurosci.

[44] Euston DR, Gruber AJ, McNaughton BL (2012). Neuron.

[45] Frankland PW, Bontempi B (2005). Nat Rev Neurosci.

[46] Kitamura T (2017). Science.

[47] Morrissey MD, Insel N, Takehara-Nishiuchi K (2017). eLife.

[48] Zhou J (2021). Nature.

[49] Lefèvre L, Gava GP (2024). Hippocampal ensemble recordings in Contextual Feeding and cNOR tasks from mice.

[50] Gava GP, Lefèvre L (2024). Analysis of hippocampal ensembles during Contextual Feeding and cNOR tasks.

[51] Perestenko P (2024). 3D printable files for objects to use in mouse object recognition tasks.

[52] Nakazawa K (2002). Science.

[53] Drane L, Ainsley JA, Mayford MR, Reijmers LG (2014). Front Mol Neurosci.

[54] Trouche S (2016). Nat Neurosci.

[55] Lopes-dos-Santos V, Brizee D, Dupret D (2023). Spatio-temporal organization of network activity patterns in the hippocampus.

[56] Han X (2011). Frontiers in Systems Neuroscience.

[57] McNamara CG, Rothwell M, Sharott A (2022). Cell Reports.

[58] Magland J (2020). Elife.

[59] Pachitariu M, Steinmetz NA, Kadir SN, Carandini M, Harris KD (2016). Advances in Neural Information Processing Systems.

[60] Lopes-Dos-Santos V (2018). Neuron.

[61] Onnela J-P, Saramäki J, Kertész J, Kaski K (2005). Physical review E, Statistical, nonlinear, and soft matter physics.

[62] Costantini G, Perugini M (2014). PLOS ONE.

[63] Saramäki J, Kivelä M, Onnela J-P, Kaski K, Kertész J (2007). Phys Rev E.

[64] Floyd RW (1962). Commun ACM.

[65] Roy B (1959). C R Acad Sci Paris.

[66] Warshall S (1962). J ACM.

[67] Estrada E (2019). Discrete Applied Mathematics.

[68] Ollion J, Cochennec J, Loll F, Escudé C, Boudier T (2013). Bioinformatics.

[69] Ho J, Tumkaya T, Aryal S, Choi H, Claridge-Chang A (2019). Nature Methods.

[70] Pedregosa F (2011). Journal of Machine Learning Research.

[71] Hagberg AA, Schult DA, Swart PJ (2008).

[72] Ince RAA, Petersen RS, Swan DC, Panzeri S (2009). Front Neuroinform.

[73] Mizuseki K, Diba K, Pastalkova E, Buzsáki G (2011). Nat Neurosci.

[74] Oliva A, Fernández-Ruiz A, Buzsáki G, Berényi A (2016). Hippocampus.

