# Supplementary material for: Organizing the coactivity structure of the hippocampus from robust to flexible memory
Source: Science. Author manuscript; Available in PMC 2024 Sep 11. (PMC7616439; doi:10.1126/science.adk9611)
Supplement: Supplementary Material [file EMS198384-supplement-Supplementary_Materials.docx]

Supplementary Materials for

**Organizing the coactivity structure of the hippocampus from robust to flexible memory**

Authors: Giuseppe P. Gava, Laura Lefèvre, Tabitha Broadbelt, Stephen B. McHugh, Vítor Lopes-dos-Santos, Demi Brizee, Katja Hartwich, Hanna Sjoberg, Pavel V. Perestenko, Robert Toth, Andrew Sharott, and David Dupret.

Corresponding author: [david.dupret@bndu.ox.ac.uk](mailto:david.dupret@bndu.ox.ac.uk)

**Includes:**

Materials and Methods

Figs. S1 to S20

Materials and Methods

**Animals.** These experiments used adult (4–6 months old) C57BL/6J wild-type mice (Charles River Laboratories, UK), transgenic hemizygous Grik4-Cre mice (The Jackson Laboratories; C57BL/6-Tg(Grik4-cre)G32-4Stl/J, stock number 006474, RRID: IMSR_JAX:006474; (*52*)) and double-transgenic mice obtained by crossing transgenic mice heterozygous for the transgene expressing the Cre recombinase under the control of the Calbindin 1 (Calb1) promoter (Jackson Laboratories; obtained from C57BL/6J crossed with Calb1-IRES2-Cre-D B6.129S-Calb1^tm2.1(cre)Hze^/J, stock number 028532, RRID: IMSR_JAX:028532) with c-fos–tTA transgenic male mice heterozygous for the transgene carrying the c-fos promoter–driven tetracycline transactivator (tTA) (*53*, *54*). The c-fos-tTA (a.k.a. TetTag) mouse line was generated at The Scripps Research Institute and obtained from Dr. L.G. Reijmers at Tufts University under a material agreement with The Scripps Research Institute for shipment to the MRC BNDU at the University of Oxford where they were bred from c-fos-tTA mice crossed with C57Bl6/J mice. Animals were housed with their littermates up until the start of the experiment. All mice held in IVCs, with wooden chew stick and nestlets in a dedicated housing facility with a 12/12 h light/dark cycle (lights on at 07:00), 19–23°C ambient temperature and 40–70% humidity. They had free access to water and food *ad libitum* throughout the experiment. Experimental procedures performed on mice in accordance with the Animals (Scientific Procedures) Act, 1986 (United Kingdom), with final ethical review by the Animals in Science Regulation Unit of the UK Home Office.

**Surgical procedure.** All surgical procedures were performed under deep anesthesia using isoflurane (0.5–2%) and oxygen (2 l/min), with analgesia provided before (0.1 mg/kg vetergesic) and after (5 mg/kg metacam) surgery.

For CA3 optogenetic silencing, we bilaterally transduced the dorsal CA3 of Grik4-Cre mice with a Cre-dependent ArchT-GFP viral construct (200 nl per site; 2 injection sites per side; see section “Viral constructs”) using stereotaxic coordinates (site 1: −1.7 mm anteroposterior and ±1.8 mm lateral from bregma, and −1.8 mm ventral from dura; site 2: −2.3 mm anteroposterior and ±2.6 mm lateral from bregma, and −2.1 mm ventral from dura). The viral vector was delivered at a rate of 100 nl/min using a glass micropipette lowered to the target site and held in place for 5 min after virus delivery before being withdrawn. Likewise, we injected a Cre-dependent GFP-only construct in the dorsal CA3 of other Grik4-Cre mice to generate the corresponding controls.

To generate expression of either ArchT-EYFP or EYFP-only in principal cells of the CA1 superficial *pyramidale* sublayer, we combined Cre-dependent, FlpO-dependent, and tTA-dependent approaches (see section “Viral constructs”). We mixed in a 1:5 ratio a tTA-inducible AAV carrying a TRE3G-FlpO construct with a Cre-dependent FlpO-dependent AAV carrying either a hSyn-Con/Fon-ArchT-EYFP or the corresponding control hSyn-Con/Fon-EYFP. Viral injections were targeted bilaterally to dorsal CA1 hippocampus of double-transgenic Calb1-Cre;cFos-tTA mice using stereotaxic coordinates (-1.7 and -2.3 mm anteroposterior from bregma, ±1.25 and ±1.7 mm lateral from bregma, and -1.1 mm ventral from dura; 150 nl per site; 2 injection sites per side) at a rate of 100 nl/min using a glass micropipette lowered to the target site and held in place for 5 min after virus delivery before being withdrawn.

For electrophysiological tetrode recordings, mice were implanted with a single microdrive containing 14 independently movable tetrodes, targeting the *stratum pyramidale* of the dorsal CA1 hippocampus (*15*). Tetrodes were constructed by twisting together four insulated tungsten wires (12 μm diameter, California Fine Wire) which were briefly heated to bind them together into a single bundle. Each tetrode was loaded in one cannula attached to a 6 mm long M1.0 screw to enable its independent manipulation of depth. The drive was implanted under stereotaxic control in reference to bregma using central coordinates -2.0 mm anteroposterior from bregma, +1.7 mm lateral from bregma as a reference to position each individual tetrode contained in the microdrive, initially implanting tetrodes above the pyramidal layer (-1.0 mm ventral from dura). The distance between neighboring tetrodes was 350 μm. Following the implantation, the exposed parts of the tetrodes were covered with paraffin wax, after which the drive was secured to the skull using dental cement and stainless-steel anchor screws inserted into the skull. Two of the anchor screws, both above the cerebellum, were attached to a 50 µm tungsten wire (California Fine Wire) and served as ground. For the recordings, each tetrode was lowered along the vertical axis to reach the pyramidale layer using the rotations applied to its tetrode cannula-holding screw and the electrophysiological profile of the local field potentials in the hippocampal ripple frequency band, with final depth position subsequently confirmed by histology of anatomical tracks.

For optogenetic manipulations, two optic fibers (230 μm diameter, Doric Lenses, Canada) were incorporated into the 14-tetrode microdrive designed to bilaterally deliver light to dorsal CA1 (or CA3 in Grik4-Cre mice) and implanted 10 days after viral injections.

To identify the location of CA1 principal cells in the deep versus the superficial sublayers of the CA1 *stratum pyramidale*, we leveraged silicon probe recordings with known spacing between the recording sites along a linear shank (*55*). Silicon probes were implanted following the same surgical procedure to span the somato-dendritic axis of CA1 principal cells and establish the laminar profile of the sharp-wave ripples detected in the local field potentials. These silicon probe recordings allowed estimating the position (depth) of individual tetrode-recorded principal cell soma with respect to the deep versus the superficial sublayers of the dorsal CA1 *stratum pyramidale* (fig. S13).

For the hippocampal lesion surgery (fig. S2C), mice in the lesion group underwent the same anesthetic induction as above, then scalp incision and craniotomy, followed by N-methy-D-aspartate (NMDA; 10 mg / mL) injections directly into the hippocampus at four sites per hemisphere using a modified Hamilton 36G syringe needle (anterior-posterior: -1.7, -2.3, -2.8, -3.1; mediolateral: ±1.2, ±1.7, ±2.2, ± 2.8; dorsoventral: -1.9, -1.9, -2.0, -4.0, respectively; all from bregma; 100 to 200 nL per site at the infusion / diffusion rates described above), and were then sutured. Midazolam (5 mg / kg, sub-cutaneous) was used to prevent seizures in hippocampal-lesioned mice. Mice receiving sham surgery were incised and then sutured. All mice had at least 2 weeks recovery before behavioral testing.

**Viral constructs.** To optogenetically target CA3 neurons genetically defined to express Grik4, we either injected the Cre-dependent AAV9-CAG-Flex-ArchT-GFP viral vector (UNC Vector Core, #AV6222b) to generate CA3^Grik4^::ArchT mice, or the corresponding AAV9-CAG-Flex-GFP control vector (UNC Vector Core, #AV5220C) to generate CA3^Grik4^::GFP mice.

To optogenetically target cFos-expressing CA1 superficial *pyramidale* sublayer neurons genetically defined to express Calbindin 1, we first produced a TRE3G-FlpO AAV carrying the optimised FlpO recombinase under the control of the third generation of tetracycline responsive element containing promoter (TRE3G, Clontech Laboratories). For this, we exchanged ArchT-GFP open reading frame (ORF) (cut with NcoI and EcoRV) in pAAV-Tre3G-ArchT-GFP vector (*54*) with the Myc-FLPo ORF (cut with NcoI and Klenow blunted AscI) from pAAV-EF1a-DIO-FLPo-Myc vector (*42*) (Addgene plasmid # 124641; http://n2t.net/addgene:124641; RRID:Addgene_124641).

We next produced a viral vector allowing the Cre-dependent and Flp-dependent expression of ArchT-eYFP. The corresponding pAAV-hSyn-Con/Fon-ArchT-eYFP construct was cloned in two stages. First, pAAV-hSyn-Coff/Fon-ArchT-eYFP has been cloned. Plasmid vectors pAAV-CamKII-ArchT-GFP (a gift from Edward Boyden, Addgene plasmid #37807; http://n2t.net/addgene:37807; RRID: Addgene_37807)(*56*), pAAV-hSyn-Coff/Fon-hChR2(H134R)-eYFP (a gift from Karl Deisseroth, Addgene plasmid #55648; http://n2t.net/addgene:55648; RRID: Addgene_55648) (*36*) and the combination of the PCR products was used to assemble two inserts that were then subcloned into the pAAV-hSyn Coff/Fon hChR2(H134R)-eYFP vector to substitute the corresponding hChR2-eYFP coding exons with the ArchT-eYFP ones. Primers and the template plasmid DNA for the first insert (exon 1): GTTTCTGCTAGCAACCCCGACACTTACCTTAGCCAGCAGGGCCAG, GTTTCTGAGCTCGCCACCATGGACCCCATC, plasmid#37807. The PCR product was then cloned into the plasmid #55648 using NheI and SacI recognition sites thus forming the intermediate vector. For the following subcloning of the exon 2 the three PCR products were generated with the primers and the corresponding template DNAs: GTTTCTACTAGTCCTCCTGTACTCACC, GTGAGCAAGGGCGAGGAG, plasmid #55648; CTCCTCGCCCTTGCTCACTGCTACTACCGGTCGGGG, GACTCTATTTCTCATGTGTTTAGGTGGACAGGGTGAGCATCG, plasmid #37807; CCTAAACACATGAGAAATAGAGTC, CGAAGTTATGGTACCTGTGCCCCCCC, plasmid #55648. These three products were then combined in the overlapping PCR and inserted into the intermediate vector using SpeI and KpnI cloning sites forming pAAV-hSyn-Coff/Fon-ArchT-eYFP. pAAV-hSyn-Coff/Fon-ArchT-YFP vector then was used to produce pAAV-hSyn-Con/Fon-ArchT-eYFP by inverting the sequence containing part of ArchT-eYFP exon and flanked with the SpeI and KpnI restriction enzymes. The corresponding insert was produced by the PCR with the primers GTTTCTACTAGTTGTGCCCCCCCTTTTTTTTAT and GTTTCTGGTACCCCTCCTGTACTCACCTTGCC using pAAV-hSyn-Coff/Fon-ArchT-eYFP vector as a template.

The control vector expressing eYFP in the Cre-dependent and Flp-dependent manner was a gift from Karl Deisseroth (Addgene plasmid # 55650; http://n2t.net/addgene:55650; RRID:Addgene_55650) (*36*).

We mixed in a 1:5 ratio the AAV carrying TRE3G-FlpO and one of the two Cre-dependent Flp-dependent AAVs (for ArchT-EYFP versus EYFP-only expression) in the CA1 of double-transgenic Calb1-Cre;cFos-tTA mice or their mono-transgenic (i.e., Calb1-Cre and c-fos–tTA) control mice (Fig. 5 and fig. S16).

**Contextual feeding task.** Following full recovery from the surgery, each mouse was first handled in a dedicated handling cloth and connected to the recording system to be familiarized with the recording procedure over a period of one week prior to the start of the experiment itself. During this period, tetrodes were gradually lowered to the CA1 *stratum pyramidale* using their estimated depth location, local field potentials, and neuronal spike waveforms. All mice were fed *ad libitum* throughout the entire experiment. Our contextual feeding task involved three open-field arenas (referred to as context *X*, *Y*, and *Z*) that were all novel environments for the mouse on conditioning day 1. These enclosures (46 cm width; 38 cm height) differed in their shape (Fig. 1A and fig. S1B, D) and wall-attached cue cards. The first stage of this task (“Food-context conditioning”) spanned either 10 (Fig. 1B) or 2 (fig. S10) consecutive days. On each conditioning day, mice explored the arenas in a random order (30-min exploration each). Context *X* contained two food containers, each provided with one regular diet pellet (Chow; fig. S1A), identical to those present in the mouse homecage. Context *Y* contained two similar food containers that allowed a choice between one chow pellet versus one high-fat diet pellet (fig. S1A; 45% Hfd; Research Diets Inc.; catalog number #D12451). Each mouse provided one data point showing its food intake per conditioning day in context *X* and context *Y*. Context *Z* did not contain food during conditioning. On each conditioning day, mice were allowed to rest for 30 minutes between two arena explorations. For the histological assessment of cFos expression, we either exposed mice to context *Y* without food, context *Y* with Hfd, or their homecage with Hfd (all 30-min experience every day for 10 days; Fig. 4A and fig. S12).

The second stage of this task (“Novel food test”) allowed probing discriminative food-context association while assessing changes in baseline population-level activity following the 10-day (versus 2-day) conditioning stage (Fig. 1 and fig. S10). For this, we first re-exposed mice to either context *X* or *Y* (“Re-exposure”; 30-min) before we then measured mouse propensity to express context-biased feeding (“Novel food test”; 30-min). In each novel food test, the arena contained two containers that allowed a choice between two food items: one had a regular chow pellet, the other had a never-seen-before resource (e.g., apricot or blackcurrant fruit jams; chocolate spread or Biscoff food pastes; fig. S1C). The energy value and palatability of novel food resources were matched across test days and contexts. Novel food intake was measured as the novel food kCal intake in context *Y* minus that in context *X*. Each mouse provided one pair of data points showing its food intake in context *Y* versus *X* per novel food test day. We further allowed mice to eat Hfd in context *Z* that then contained two food containers (one with a chow pellet, the other with a Hfd pellet) on some cNOR days (“Hfd test”). We first used this to assess the contextual Hfd feeding bias of each mouse by measuring its Hfd intake over 30 minutes in context *Z* minus that measured in context *Y* in the previous day, over their sum (fig. S1D). We also used this Hfd test in context *Z* before engaging mice in the cNOR task to evaluate whether they would express a defective object-location memory in context *Y*, versus a successful object-location memory in context *X*, regardless of whether they were fed or not with Hfd just before that day (fig. S3).

We adapted our contextual feeding paradigm in Calb1-Cre;cFos-tTA mice to optogenetically tag the set of CA1 superficial *pyramidale* sublayer cells that are genetically defined by Calbindin 1 and are expressing cFos with their recruitment in a given context. This activity-dependent tagging of CA1 superficial cells was performed in either the Hfd-paired context *Y* or in a fourth, neutral arena (referred to as context *W*) unrelated to the contextual feeding task (Fig. 5 and fig. S17). For this, Calb1-Cre;cFos-tTA mice were fed for 10 days with doxycycline-containing pellets (Envigo Ltd., Catalog number #TD120240) prior to viral construct injections and microdrive implantation (fig. S17A). In this set of experiments, we replaced the Chow pellets with Dox pellets in the mouse homecage and both context *X* and *Y*, except when conducting the neuronal tagging during the off-Dox days in context *Y* versus *W*, as appropriate (fig. S17A). Before these tagging days, we conducted the first 2 days of conditioning in context *X* (fig. S17A). For the optogenetic tagging, we then ceased the dox-containing pellet homecage feeding for two days when we exposed mice to either context *Y* or *W* (fig. S17A), after which they received high-doxycycline containing pellets (Envigo Ltd., Catalog number #TD120658) as homecage feeding for 24 hours before returning to normal doxycycline containing pellets for the remaining of the experiment.

**continuous Novel Object Recognition task.** On each cNOR task day (Fig. 1B and figs. S2A, S10A, S11A, S17A), mice first re-explored context *X* or *Y* (“Re-exposure”; without any food; 15-min exploration). We used these re-exposure sessions to evaluate enduring changes in baseline population-level activity following food-context conditioning. In this context, we next inserted four novel objects (“Sampling”; 15-min exploration). Mice continued to explore this context for four more sessions (“Tests”; 10-min exploration each) that day (figs. S2A, S10A, S11A, S17A). Each object (outer dimension: 50 x 50 mm width; 55 mm height; example objects shown in fig. S2B) was positioned midway along a given wall. From the sampling session to the first cNOR test and then for each other cNOR test, we replaced one of the objects initially sampled with a novel object. This procedure thus allowed the mouse to explore three familiar (previously seen) and one completely novel object on each cNOR test that day. On each test, we measured the time spent exploring each object and we calculated the percentage time spent with the novel object versus the (mean) percentage time spent with the familiar objects. Each mouse provided one data point showing its time spent exploring a given object per cNOR test. The interval between two cNOR test sessions was 5-min, during which the mouse was in a sleep/rest box temporally placed in the recording arena. Mice could eat regular-diet (Chow) food before each cNOR day experiment, as any other day of our paradigm to ensure they would not perform tests while hungry. We finished each cNOR day by re-conditioning mice, exposing them again to context *Y* with Hfd (30-min session).

**Multichannel data acquisition, position tracking and light delivery.** The extracellular signals from each tetrode channel were amplified, multiplexed, and digitized using a single integrated circuit located on the head of the animal (RHD2164, Intan Technologies; <http://intantech.com/products_RHD2000.html>; pass band 0.09 Hz to 7.60 kHz). The amplified and filtered electrophysiological signals were digitized at 20 kHz and saved to disk along with the synchronization signals (transistor-transistor logic digital pulses) reporting the animal’s position tracking and laser activations. The location of the animal was tracked using three differently colored LED clusters attached to the electrode casing and captured at 39 frames per second by an overhead color camera (<https://github.com/kevin-allen/positrack/wiki>). The LFPs were down-sampled to 1,250 Hz for all subsequent analyses. For optogenetic intervention, a 561-nm diode-pumped solid-state laser (Crystal Laser, model CL561-100; distributer: Laser 2000, Ringstead, UK) was used to feed implanted optic fibers with light (~15–20mW input power; ~9–11mW output power) bilaterally to the dorsal CA3 (Fig. 3) or CA1 (Fig. 5) hippocampus via a 2-channel rotary joint (Doric Lenses Inc.). This was performed using a closed-loop system to deliver in real time 561-nm light 50-ms pulses using dynamic tracking of ongoing theta phase in CA3^Grik4^::ArchT mice and CA3^Grik4^::GFP mice (Fig. 3 and fig. S11), CA1^Calb1-cFos^::ArchT mice and CA1^Calb1-cFos^::EYFP mice (Fig. 5 and fig. S18). The real-time phase estimate was obtained using the OscillTrack algorithm (*57*) implemented in the field-programmable gate array of the Intan Technologies interface board. Phase detection was obtained by continuously operating on the data stream coming from an input channel containing the CA1 pyramidal layer LFPs. This input channel used as the phase reference was high-pass filtered using a 1st order digital infinite impulse response filter with a corner frequency of 0.4 Hz to remove amplifier offset and electrode drift, then down-sampled 125-fold to a rate of 160 Hz for processing. The phase estimation was operated with a loop-gain of 0.0625 at a center frequency of 7 Hz. Stimulation was triggered with a phase lead to align the target phase with the middle of the 50-ms light pulse.

**Spike detection and unit isolation.** Spike sorting and unit isolation were performed with an automated clustering pipeline using Kilosort (<https://github.com/cortex-lab/KiloSort>) via the SpikeForest framework (<https://github.com/flatironinstitute/spikeforest>) (*58*, *59*). To apply KiloSort to data acquired using tetrodes, the algorithm restricted templates to channels within a given tetrode bundle, while masking all other recording channels. All sessions recorded on a given day were concatenated and cluster cut together to monitor cells throughout the experiment day. The resulting clusters were verified by the operator using cross-channel spike waveforms, auto-correlation histograms, and cross-correlation histograms. Each unit used for analyses showed throughout the entire recording day stable spike waveforms, clear refractory period in their auto-correlation histogram, and absence of refractory period in its cross-correlation histograms with the other units. The trough-to-peak width of the spike waveform was used to distinguish hippocampal principal cells from interneurons, as described previously (*55*). In total, this study includes 9,111 CA1 principal cells: 2,506 using 6 wild-type mice for the original 10-day conditioning dataset (Figs. 1, 2, and 4); 1,389 using 5 wild-type mice in the additional 2-day conditioning dataset (fig. S10); 1,548 using 4 CA3^Grik4^::ArchT mice and 881 using 2 CA3^Grik4^::GFP control mice for the CA3 silencing dataset (Fig. 3); 1,097 using 5 CA1^Calb1-cFos^::ArchT mice tagged with ArchT in context *Y*, 1,007 using 4 CA1^Calb1-cFos^::EYFP mice tagged with EYFP in context *Y*, and 683 using 3 CA1^Calb1-cFos^::ArchT mice tagged with ArchT in context *W* for the optogenetic tagging dataset (Fig. 5).

**Theta oscillations, sharp-wave ripples detection, and tetrode depth estimation.**

To detect theta oscillations from the LFPs, we applied Ensemble Empirical Mode Decomposition and selected bouts of at least 5 cycles during active exploratory behavior (animal speed > 2 cm/s) (*60*). To detect sharp-wave ripples, the LFP signal of each CA1 pyramidal layer channel was subtracted by the mean across all channels/sites (common average reference). These re-referenced signals were then filtered for the ripple band (110 to 250 Hz; 4th order Butterworth filter) and their envelopes (instantaneous amplitudes) were computed by means of the Hilbert transform. The peaks (local maxima) of the ripple band envelope signals above a threshold (5 times the median of the envelope values of that channel) were regarded as candidate events. Further, the onset and offset of each event were determined as the time points at which the ripple envelope decayed below half of the detection threshold. Candidate events passing the following criteria were determined as SWR events: (*i*) ripple band power in the event channel was at least 2 times the ripple band power in the common average reference (to eliminate common high frequency noise); (*ii*) an event had at least four ripple cycles (to eliminate events that were too brief); (*iii*) ripple band power was at least 2 times higher than the supra-ripple band defined as 200-500 Hz (to eliminate high frequency noise, not spectrally compact at the ripple band, such as spike leakage artefacts). We estimated the tetrode depth within the pyramidal layer using SWR and theta waveforms, as previously described (*55*). Raw local field potentials of each tetrode were averaged around the SWR power peaks for sharp-wave waveforms and around the descending zero crossings of theta oscillations for theta waveforms. The channel with the highest ripple power was used as theta phase reference. These waveforms were then projected onto a feature embedding template created using silicon probe recordings (*55*). Deep sublayer tetrodes (closer to oriens) showed positive sharp-waves, while superficial ones (closer to radiatum) displayed negative sharp-waves (fig. S13). Additionally, superficial channels exhibited stronger theta-nested slow-gamma components.

**Object-location population decoding.** We trained a General Linear Model (GLM) to classify each object-location compound explored during cNOR tests, from the recorded neuronal spiking activity nested in theta cycles associated with these visits (Fig. 1F). That is, we used the population vectors of action potentials discharged by CA1 principal cells in the theta cycles associated with the mouse active exploration of each object-location compound. In doing so, the animal’s body was within a radius of 5 cm from the object being explored. By training a model in session $n-1$ and testing it in test $n$ we obtained one novel and three familiar object scores per cNOR session (four session pairs per day; e.g., Fig. 1G).

A given object-location compound pair $x$ was then associated to a population decoding vector composed of each neuron’s GLM coefficient ($P^{x}$), reporting the individual weight contribution of neuron $i$ to the population representation of $x$ (Fig. 1H). By taking the cosine distance metric between pairs of decoding vectors across subsequent sessions representing the same location but different objects (familiar object $x$ versus novel object $y$), we quantified the change in population representation $D_{pop}=dist_{cos}\left( P^{x}, P^{y} \right)$ (decoding vectors similarity (Fig. 1I). The representational change of individual neurons induced by the insertion of novel objects, was instead computed as $d_{i}^{xy}=abs\left( c_{i}^{x}-c_{i}^{y} \right)$ (referred to as “$\Delta$single-neuron decoding coefficient”, Fig. 1J).

To build the GLM model, we employed the function *LogisticRegression* from the *sklearn* package (v .1.2.2) with L2 regularization (inverse strength *C*=1e-2, selected by maximizing the classification scores across sessions).

**Spatial rate maps.** The horizontal plane of the recording arena was divided into bins of 2 × 2 cm to generate spike count maps (number of spikes fired in each bin) for each unit and an occupancy map (time spent by the animal in each bin), considering only active exploratory behavior (speed>2 cm.s-1), discarding periods of immobility, and further excluding sharp-wave/ripples. Then, spatial rate maps were generated by normalizing (dividing) the spike count maps by the corresponding occupancy map. The spatial rate maps were smoothed by convolution with a Gaussian kernel having s.d. equal to one bin width.

**Single-neuron place field similarity.** In this study, the place field similarity (PFS) is a pairwise measure that compares the place maps expressed by a given cell across two contiguous cNOR sessions (e.g., sampling versus test 1) in a given context. It represents the degree to which a cell that fired in a given region of space during one session (e.g., sampling) still fired in that region of space during the subsequent session (e.g., test 1). For each cell, the PFS value for a given pair of cNOR sessions is computed as the Pearson correlation coefficient from the direct bin wise comparisons between its two spatial rate maps (non-smoothed place maps; one map per session), limited to valid bins (occupancy greater than zero).

**Neuronal coactivity graphs.** We constructed hippocampal population graphs that represent the coactivity relationships between all pairs of principal cell spike trains recorded during a given task session. These coactivity graphs were computed using theta cycles as time windows spanning active exploratory behavior (speed>2 cm.s-1), discarding periods of immobility and further excluding sharp-wave/ripples. To further control for the shared influence of the general network activity on peer-to-peer coactivity, we used for any two neurons $(i,j)$ the regression coefficient $\beta$ obtained by fitting the GLM (Fig. 2C):

$$x_{j}\sim\beta_{ij}x_{i}+\alpha_{ij}P$$

where $x_{j}, x_{i}$ are the z-scored theta-nested spike trains of individual neurons *j* (the target) and *i* (the predictor), and $P$ is the summed activity of the other $N-2$ neurons,

$$P=\sum_{n=0}^{N-\left\{ i, j \right\}} x_{n}$$

with $\alpha_{ij}$ weighting the influence of the population contribution to the activity of target neuron *j.*

The recorded neurons (and their coactivity associations) are therefore the nodes (and their edges) in the coactivity graph of each task session. We described each graph by its adjacency matrix, *A*, as the *N*x *N* square matrix containing the pairwise coactivity relations within the network, yielding a weighted graph with no self-connections:

$$A=\left( \begin{matrix} \beta_{0,0} & \cdots& \beta_{0,N} \\ \vdots& \ddots& \vdots\\ \beta_{N,0} & \cdots& \beta_{N,N} \end{matrix} \right)$$

with $\beta_{i,i}=0 \forall i in N$, and the symmetry in the weights of the network being ensured by setting $A=\frac{A+A^{T}}{2}$ to form an undirected graph.

**Clustering coefficient.** We computed the clustering coefficient $C_{i}$ to characterize the network’s local coactivity structure by scoring the triadic firing relationships established by each neuron *i* with the other neurons in the population, using the formula proposed by Onnela et al. (*61*–*63*):

$$C_{i}=\frac{\sum_{jq} \left( \hat{\beta_{ij}}\hat{\beta_{iq}}\hat{\beta_{jq}} \right)^{1/3}}{k_{i}\left( k_{i}-1 \right)}$$

where *j* and *q* are neighbors of neuron *i*, all edge weights are normalized by the maximum edge weight in the network $\hat{\beta} =\beta/\max\left( \beta\right)$, and *k_i_* is the degree of neuron *i*, which in these weighted graphs with no self-connection is equal to the number of neurons minus one. This formula accounts for negative edges, yielding a negative value when there is an odd number due to the negative edges in the triad; it is positive otherwise. For this reason, this quantity can also be interpreted as a measure of the structural balance around a node, in the sense that its neighbourhood presents coherent patterns of firing relationships (see below).

**Geodesic path length.** We measured the geodesic (i.e., shortest) path length to estimate the ability of the network to efficiently switch from the activity of one node to another (Fig. 2F). In a binary graph, this functional measure would represent the smallest number of edges connecting two nodes. Here, we define the length between two nodes *i* and *j* as the inverse of their coactivity: $l=1/\beta_{ij}$, discarding all negative edges (*18*). We then computed the geodesic path length between any two nodes in each neuronal graph using the Floyd-Warshall algorithm (*64*–*66*). We report the average path length for each principal cell in the coactivity graph to evaluate the easiness of transitioning across nodes in the hippocampus network.

**Single-neuron coactivity strength.** We defined the single-neuron coactivity strength as the average pairwise coactivity relation of a given node in a weighted graph (fig. S5B). As a reference, the strength in a weighted graph can be compared to the degree in a binary graph, which accounts for the number of the node’s neighbors. Here, the strength $S_{i}$ of a node *i* is the average across all the weights $\beta_{ij}$ of the edges projected from that node:

$S_{i}=\frac{\sum_{j=0}^{N} \beta_{ij}}{N}$

where *N* is the number of neurons *j* that node *i* projects to.

**Null models of coactivity graphs.** To characterize further the structure of coactivity graphs, we compared them with equivalent null models of known topology, namely their random and lattice surrogates (fig. S6A). For each real-data neuronal graph, we aimed to construct a random equivalent graph where the average geodesic path length would be shorter while the original distribution of the observed coactivity strength value of each node would be preserved. Interestingly, we noted that the control graphs obtained by randomly shuffling the edge weights of the real-data coactivity graphs however displayed longer geodesic path lengths than those of the real-data graphs (*18*). Thus, we randomly reassigned the edges of each neuronal node to preserve its coactivity strength by first extracting the upper-triangular matrix $U$of the coactivity graph $A$, then row-wise shuffle $U$to obtain $U_{r}$ and construct the random equivalent graph $R = U_{r}+U_{r}^{T}$ where $R$is the random equivalent graph of $A$that preserved the original single-neuron coactivity values.

For each real-data neuronal graph, we also constructed a lattice equivalent graph by re-arranging its original coactivity edge weights onto a one-dimensional (circular) lattice, such that the edges that corresponded to the smallest Euclidean distance between nodes were assigned the highest weights. We reasoned that in a 1-D lattice with $N$nodes and unit spacing between nodes, there are *N* edges with a Euclidean distance of $d=1$ between nodes. We therefore ranked the observed edge weights by decreased strength and randomly distributed the top $N$connections among the edges representing $d=1$, the next $N-1$weights to the $d=2$edges, etc. We continued in this manner until all the edges in the original coactivity graph were placed in the lattice (*18*).

**Structural balance.** The combination of positive and negative edges in a network can give rise to both stable and unstable patterns of relationships. We applied structural balance analysis to neuron coactivity relationships to assess their coherence, and therefore the stability of hippocampal graphs. In this network analysis, triads of neurons are classified as balanced or unbalanced (fig. S6G). An intuition about such triadic relationships arises from social networks where three nodes form a balanced motif either by having three positive edges (e.g., “the friend of my friend is my friend”), or by having two negative ones (e.g., “the enemy of my friend is my enemy”) (*67*). Any triad with an odd number of negative relations would make the motif unbalanced. We inspected the triadic coactivity relationships of hippocampal graphs and assessed their stability by considering for each graph the top 10% of edges with the highest absolute magnitude (both positives and negatives), finding all triads, and classifying them into balanced or unbalanced (as per the sign of their three edges). We defined the structural balance of the graph as the proportion of its balanced triads. With this, we also characterized the structure of the equivalent null models (fig. S6H). We found that random graphs are less stable, displaying a structural balance at chance level, while lattice graphs contain only balanced motifs and are therefore more stable.

**Population coupling.** We defined the coupling of neuron *i* to the rest of the population of *N* units (population coupling, *PC*) as the Pearson correlation between its theta-binned activity $x_{i}$ and that of all remaining neurons summed together $P^{N-\left\{ i \right\}}$:

$$PC_{i}=Pearson\left( x_{i}, P^{N-\left\{ i \right\}} \right)$$

To assess whether the increase in population coupling in context *Y* was due to the fine temporal structure of the neural population activity, we independently circularly shuffled the neurons’ theta-binned activity by random delays between 10 to 100 theta cycles, therefore preserving each neurons’ rate and autocorrelation. The final value was taken as the average over 100 iterations.

**Anatomy.** Mice were deeply anesthetized with isoflurane/pentobarbital and transcardially perfused with PBS followed by cold 4 % PFA dissolved in PBS. The brains were extracted, kept in 4% PFA for 24 h, and sliced into 50 µm thick coronal sections. For immunostaining, free-floating sections were rinsed extensively in PBS with 0.25 % Triton X-100 (PBS-T) and blocked for 1 h at room temperature in PBS-T with 10 % normal donkey serum (NDS). Sections were then incubated at 4°C for 48 h with primary antibodies (rabbit anti-cFos, 1:500, Synaptic Systems, cat# 226 003; goat anti-calbindin 1, 1:1,000, Nittobo Medical, cat# Af1040) diluted in PBS-T with 3 % NDS blocking solution. Sections were then rinsed three times for 10 min in PBS and incubated for 24 h at 4°C in secondary antibodies (donkey anti-rabbit Alexa Fluor 488, 1:250, Thermo Fisher Scientific, cat# A-21206, RRID:AB_2535792; donkey anti-goat Cy3, 1:1000, Jackson ImmunoResearch, cat# 705-165-147, RRID: AB_2307351) diluted in PBS-T with 1 % NDS. This step was followed by three rinses for 15 min in PBS. Sections were then incubated for 1 min with 4',6-diamidino-2-phenylindole (DAPI; 0.5 μg/ml, Sigma-Aldrich, cat# D8417) diluted in PBS to label cell nuclei, before undergoing three additional rinse steps of 10 min each in PBS. Sections were finally mounted on slides, cover-slipped with Vectashield mounting medium (Vector Laboratories) and stored at 4 °C. Sections were also used for anatomical verification of the tetrode tracks. Mice with incorrect tetrode or viral targeting were excluded. The CA1 pyramidal layer was imaged in all 3 channels (DAPI-A405, cFos-A488, and Calb1-CY3) as a mosaic stack across the full z-range using a Zeiss confocal microscope (LSM 880 Indimo, Axio Imager 2) with a Plan-Apochromat 20x/0.8 M27 Objective and the ZEN software (Zeiss Black 2.3, step size: 10μm) using 2 sections at the same rostro-caudal position (-2.0mm and -2.5 mm) for each mouse. Automated counting was performed using Fiji and the 3D ImageJ Suite (*68*). A CA1 stratum pyramidale mask was generated using the DAPI channel, subdividing it into the deep versus the superficial pyramidale sublayers using the Calbindin 1 channel. To automatically detect single cFos cells, a 3D iterative thresholding (minimum threshold: RenyEntropy, pixel-volume: 2000-3000) was used on the cFos stack. A cFos positive cell was also positive for Calb1 when the average intensity within the cFos segment was above the automatic threshold (3D intensity measure, threshold: Huang). Cell densities are number of cells divided by the volume of the pyramidale layer. For the hippocampal lesion experiment, mice were perfused transcardially with physiological saline (0.9% NaCl) followed by 10% formol saline (10% formalin in physiological saline). The brains were then removed and placed in 10% formol saline and 72 hours later transferred to 30% sucrose-formalin. Coronal sections (50 μm) were cut on a freezing microtome and stained with cresyl violet to enable visualization of lesion extent.

**Data and statistical analyses.** Data were analysed in Python 3.6 and using the packages DABEST v0.3.0 (*69*), scikit-learn v0.23.2 (*70*), NetworkX v2.4 (*71*), Numpy v1.18.1, Scipy v1.4.1, Matplotlib v3.1.2, Pandas v0.25.3 and Seaborn v0.11.0. All statistical tests related to a symmetric distribution were performed two-sided using permutation tests and visualized with Gardner-Altman plots (to compare 2 groups) and Cumming plots (for more groups) (*69*). These estimation plots allow visualizing the effect size by plotting the data as the mean or median difference between one of the groups (the left-most group of each plot, used as group-reference) and the other groups (to the right, along the x-axis of each plot). For each estimation plot: (*i*) the upper panel shows the distribution of raw data points for the entire dataset, superimposed on bar-plots reporting group mean±SD, unless stated otherwise; and (*ii*) the lower panel displays the difference between a given group and the (left-most) group-reference, computed from 5,000 bootstrapped resamples and with difference-axis origin aligned to the mean or the median of the group-reference distribution. For each estimation plot: *black-dot*, mean (for normal distributions) or median (for skewed distributions) as indicated; *black-ticks*, 95% confidence interval; *filled-curve*: bootstrapped sampling-error distribution. Data distributions were assumed to be normal, but this was not formally tested. We also used the t-test to compare two conditions; the Wald test for assessing the significance of regression lines; and the Kolmogorov–Smirnov test for comparing probability distributions. Neural and behavioral data analyses were conducted in an identical way regardless of the identity of the experimental condition from which the data were collected, with the investigator blind to group allocation during data collection and/or analysis.

**Supplementary Figures and Legends**


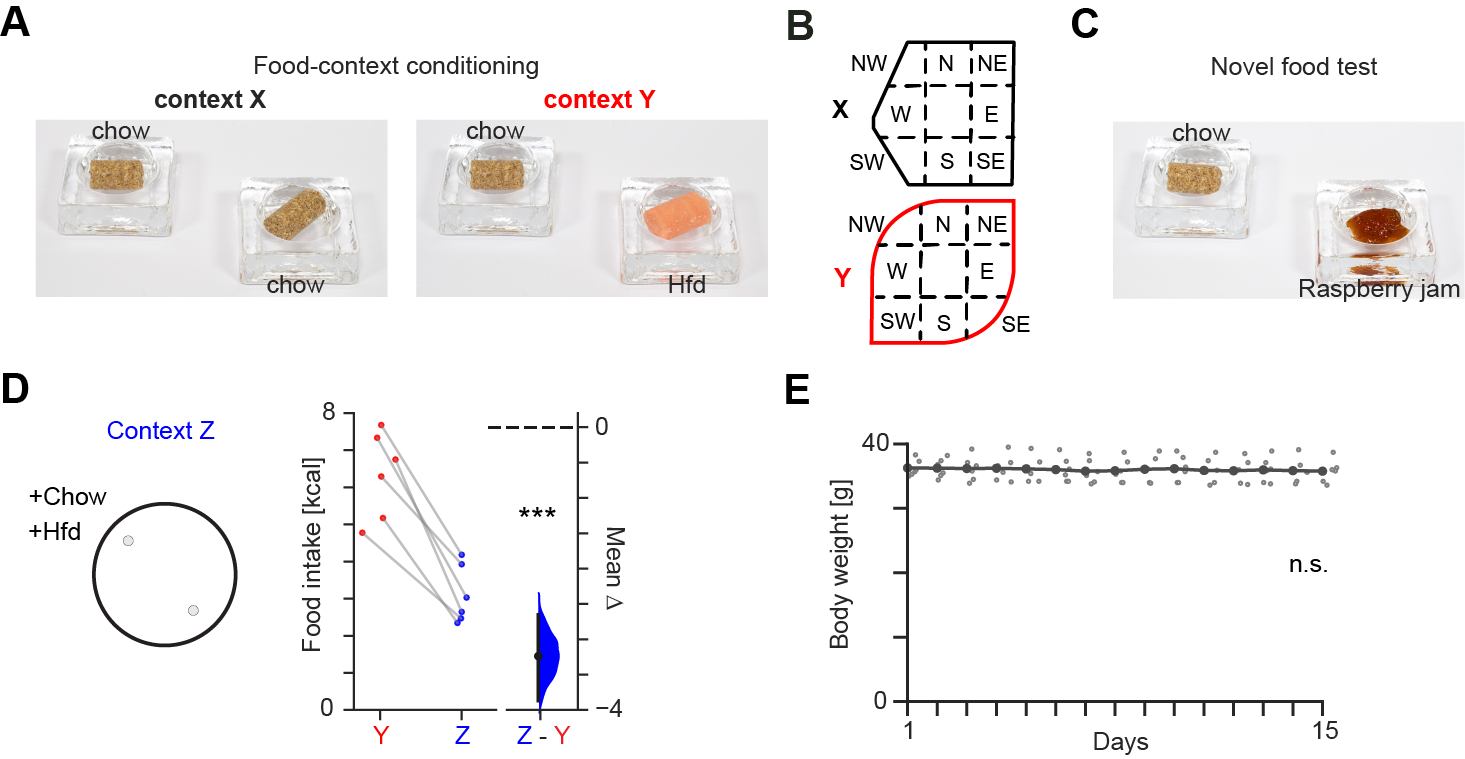


**Fig. S1. Contextual feeding task.**

**(A)** Pictures of the two food containers used to provide mice with a choice of two chow pellets in context *X* (left) and a choice between one chow pellet versus one high-fat diet (Hfd) pellet in context *Y* (right). These were used on each day of the food-context conditioning.

**(B)** Schematic of each context arena (top view) showing the eight possible food locations (cardinal and intercardinal directions: *N*, north; *NE*, northeast; *E*, east; *SE*, southeast; *S*, south; *SW*, southwest; *W*, west; and *NW*, northwest) used throughout the whole paradigm. For each behavioral session and context, we randomly selected two of these locations to position the food containers where we provided the regular-diet Chow pellets in context *X*, and the Chow versus Hfd pellets in context *Y*. We used this randomization of food locations across all behavioral sessions to promote mnemonic association of food items to whole context as opposed to any discrete place. This randomization of food locations was also applied to the novel food tests and the re-conditioning session performed at the end of each testing day (to prevent behavioral extinction).

**(C)** Shown are the two food containers used to provide mice with a choice between one chow pellet versus a new food resource (e.g., raspberry jam) during the post-conditioning novel food tests (e.g., days 11 and 12 in the 10-day conditioning paradigm).

(**D)** Layout and intake for the post-conditioning Hfd food test in context *Z*. Shown on the left is a top-view schematic of context *Z* as a circular-shaped arena that mice also explored on each conditioning day. For the post-conditioning Hfd food test, mice re-explored this neutral context *Z* now with two food containers providing a choice between one chow pellet versus one Hfd pellet (as in context *Y* during conditioning; see A). Shown on the right is the corresponding estimation plot (see methods) showing the effect size for the difference in Hfd intake between context *Y* versus context *Z*. Note the significantly stronger Hfd food intake in context *Y* compared to *Z* (p<0.001, two-sided paired permutation test).

**(E)** Mice body weight across experiment days remained stable (each data point represents one mouse; t=-0.68, p=0.5; multiple regression).

***P<0.001, two-sided paired permutation test, 6 mice.


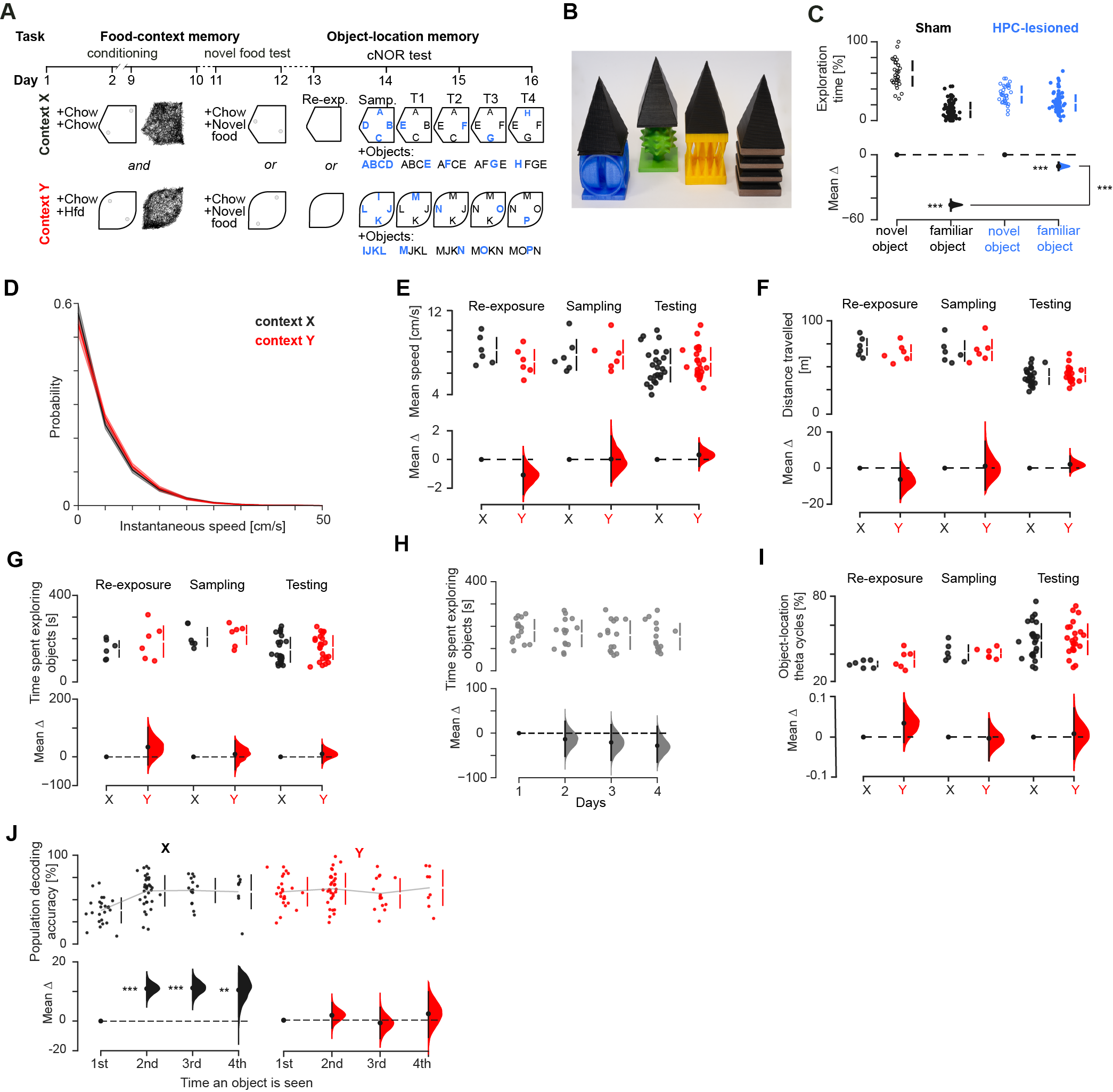


**Fig. S2. Continuous Novel Object Recognition task and cross-trial population decoding.**

(**A**) Layout of the experimental paradigm (as in Fig. 1B) with further details about the cNOR task. On each cNOR day, we first re-exposed mice to context *X* and *Y* (Re-exposure, labelled “Re-exp.”; 15-min exploration without any food; *X* and *Y* explored in random order across days) to evaluate post-conditioning changes in baseline population-level activity. Then, in the second context (*X* or *Y*) explored that day, mice explored four novel objects (Sampling, labelled “Samp.”; 15-min exploration). Each object was positioned midway along a given wall. Over four additional sessions (Tests, labelled “T1 to T4”; 10-min each), mice continued to explore this context where one of the initial objects from the sampling session was replaced by a novel one. That is, by the fourth cNOR test, all initial novel objects (e.g., A, B, C, and D) were replaced by another one (e.g., E, F, G, and H). Using this procedure, mice were exposed to three familiar (previously seen) and one completely novel object on each cNOR test. On each test, we measured the time spent on each object and we calculated the percentage time spent investigating the novel object versus the (mean) percentage time spent investigating the familiar objects.

(**B**) Example set of four objects. The “pyramid hat” on each object was used to prevent mice from climbing and staying onto the objects.

(**C**) Hippocampal lesions impair cNOR performance. Estimation plot showing the effect size in the difference in time spent exploring novel versus familiar objects. Mice with sham lesions showed a strong preference for the novel object over the familiar (p < 0.001, two-sided paired permutation test; n = 8 mice; 2 cNOR test days per mouse). Mice with hippocampal lesions showed significantly weaker cNOR performance (p < 0.001, two-sided paired permutation test; n = 8 mice; 2 cNOR days per mouse). Direct comparison showed significantly stronger novelty preference in sham compared to hippocampal-lesioned mice (p = 0.005, two-sided permutation test).

(**D-F**) Locomotor speed and distance travelled did not differ between context *X* and *Y*. This is reported in the distribution of instantaneous speed values (D); the estimation plot showing the mean animal speed during re-exposure, sampling, and test sessions (E); and the corresponding distance travelled (F).

(**G-I**) Estimation plots showing that the time spent exploring the four object locations was not different across contexts during the re-exposure, sampling and test sessions (G) and across the four cNOR days (H). Likewise, the percentage of theta cycles during exploration of each object-location was not different across contexts (I).

(**J**) Decoding accuracy of each object-location across the four cNOR tests. The 4 objects initially sampled are replaced by a new object, one at a time across tests. Thus, only one of the initially sampled objects is seen 4 times; and the last one being replaced is only seen once. Decoding increased with the number of times an object was encountered in context *X* (suggesting a gain in familiarity), but this was not the case in context *Y*.

**P<0.01, ***P<0.001, two-sided paired permutation test, 6 mice.


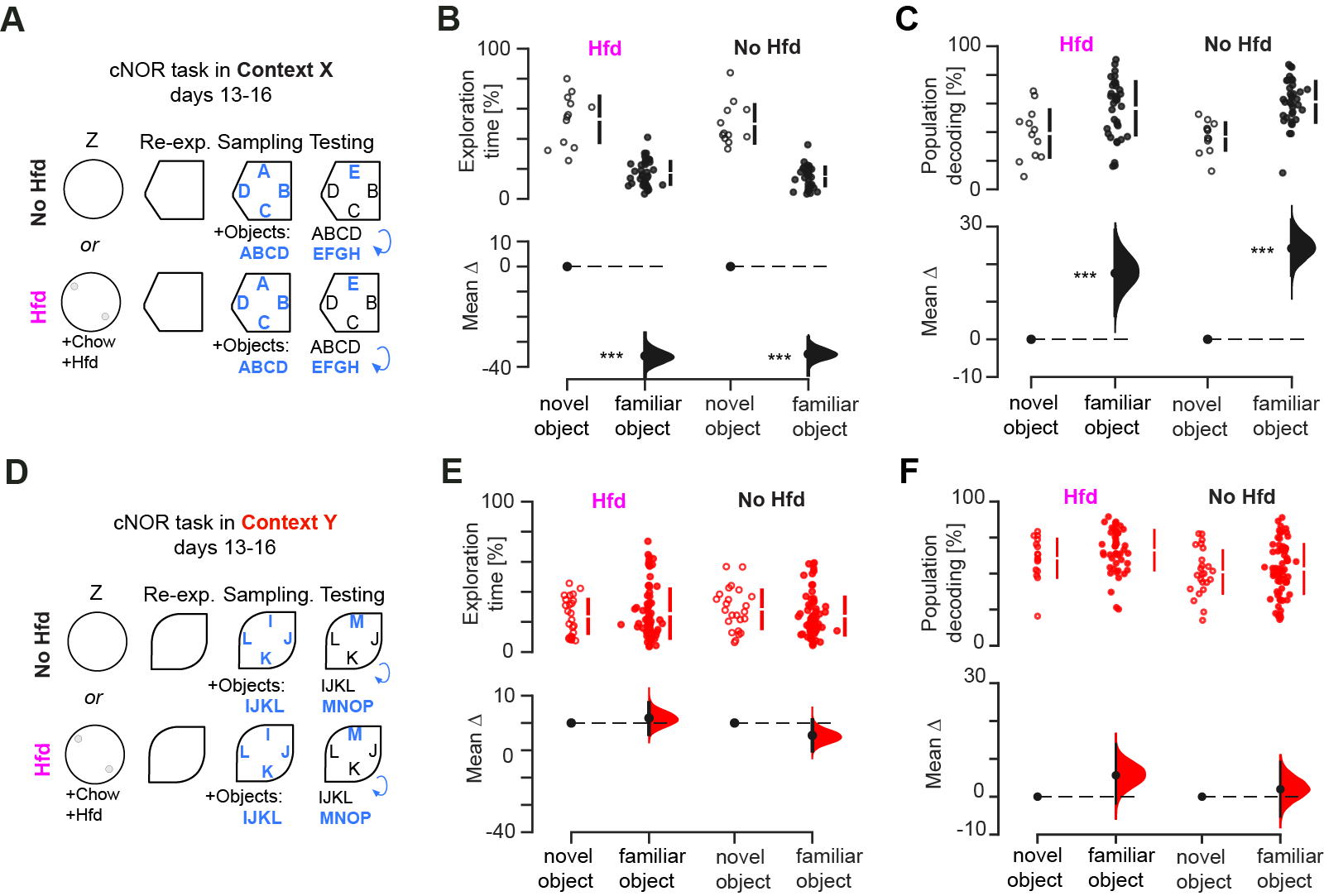


**Fig. S3. Eating Hfd prior to cNOR does not change contextual object-location memory.**

Following 10 days conditioning, mice were provided on some cNOR days with Chow and Hfd pellets in the neutral context *Z* (fig. S1D) prior to performing the cNOR task in either context *X* (A-C) or context *Y* (D-F) that day. Shown are the layouts of the corresponding sessions (A, D), the percentage of exploration time with novel and familiar objects during cNOR tests (B, E; as in Fig. 1E), and the classification accuracy of object-location compound in test $n$ by the GLM trained in session $n-1$ (C, F; as in Fig. 1G). Note that prior Hfd feeding neither changed the successful object-location memory in context *X* nor the defective one in context *Y*.

***P<0.001, two-sided paired permutation test. N = 3 mice with Hfd and 3 mice without Hfd prior to cNOR in *X* (A-C); 2 mice with Hfd and 3 mice without Hfd prior to cNOR in *Y* (D-F).


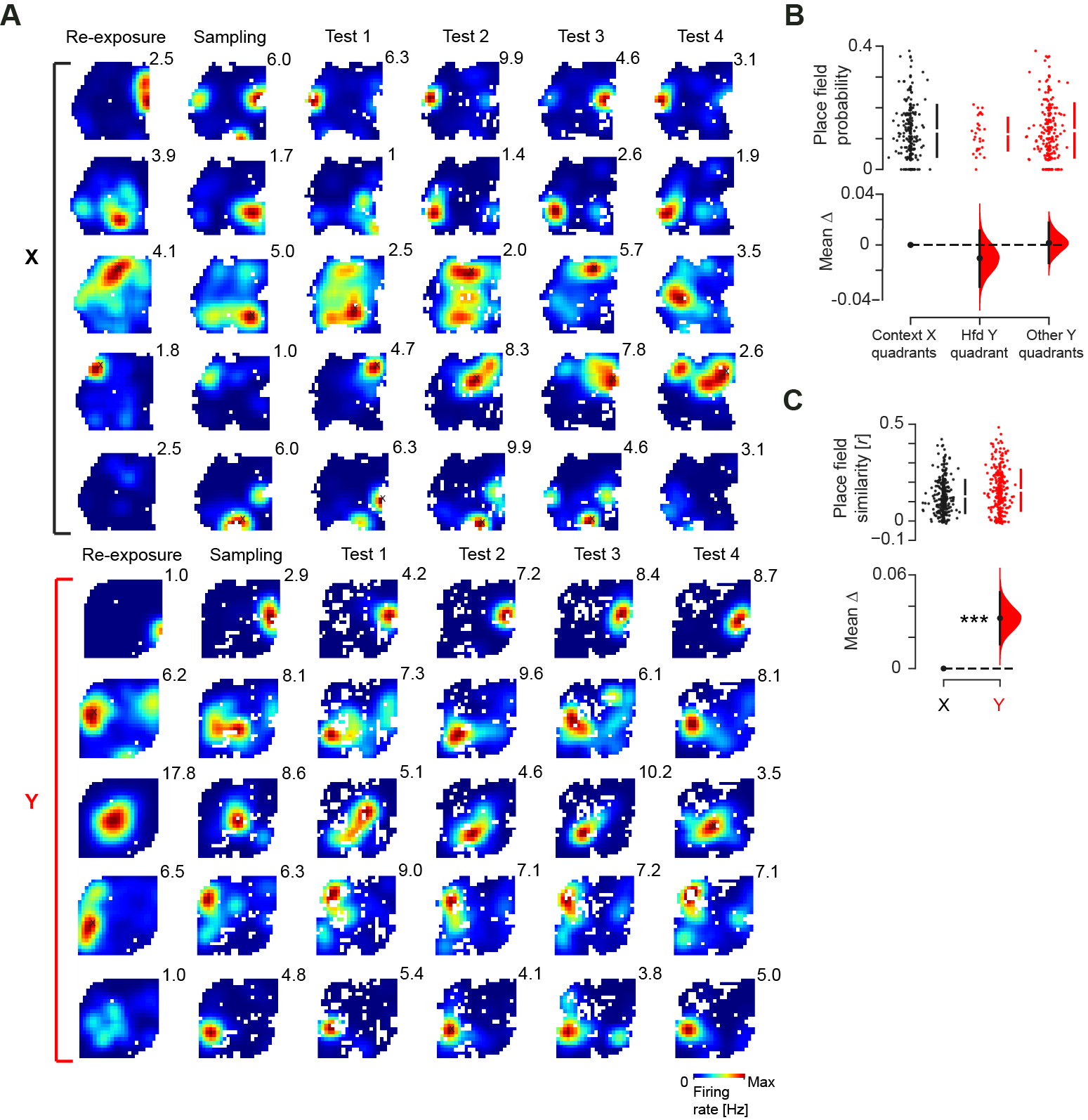


**Fig. S4. CA1 place maps are more stable across cNOR sessions in context *Y*.**

**(A)** Example CA1 firing maps across the consecutive cNOR sessions for one mouse day in context *X* (top) and one day in context *Y* (bottom). One principal cell per row (with numbers indicating peak firing rate for each map).

**(B)** The defective object-location population decoding expressed in context *Y* (Fig. 1F-J) was not explained by a CA1 place field over-representation of the last Hfd location. In our paradigm, food locations (fig. S1B) were randomized across days to promote mnemonic association of food to whole context as opposed to a fixed, discrete location. This randomization was applied on each conditioning session and on the re-conditioning session conducted at the end of each test day (to prevent extinction). Consistent with this, the probability for CA1 place fields to represent the previously experienced Hfd location while performing cNOR tests in context *Y* (“Hfd *Y* quadrant”) was not significantly different from the probability to represent any other locations in context *Y* (“Other *Y* quadrants”). This is also compared to the probability to observe place field peaks in any quadrants of context *X* where Hfd has never been provided (“Context *X* quadrants”). We found this by comparing the amount of place field peaks in the last encountered Hfd quadrant with that in the other quadrants. This indicates that no over-representation of the past Hfd location could explain the defective object-location memory (Fig. 1E) and population decoding (Fig. 1G) in context *Y*.

**(C)** Mean place field similarity (PFS) for the pairs of place maps expressed by a given CA1 principal cell across two contiguous cNOR sessions in context *X* versus those in context *Y*. Here, the results of this analysis are using the mean PFS across all pairs of contiguous cNOR sessions for each cell. Each data point represents one CA1 place cell. This PFS analysis shows that the hippocampal CA1 place code exhibited greater stability across cNOR sessions when performed in context *Y*, but stronger spatial remapping when in context *X*. See Fig. 2B for this analysis applied to the individual PFS for each pair of contiguous cNOR sessions for each place cell. ***P<0.001, two-sided paired permutation test, 6 mice, 2506 CA1 principal cells.


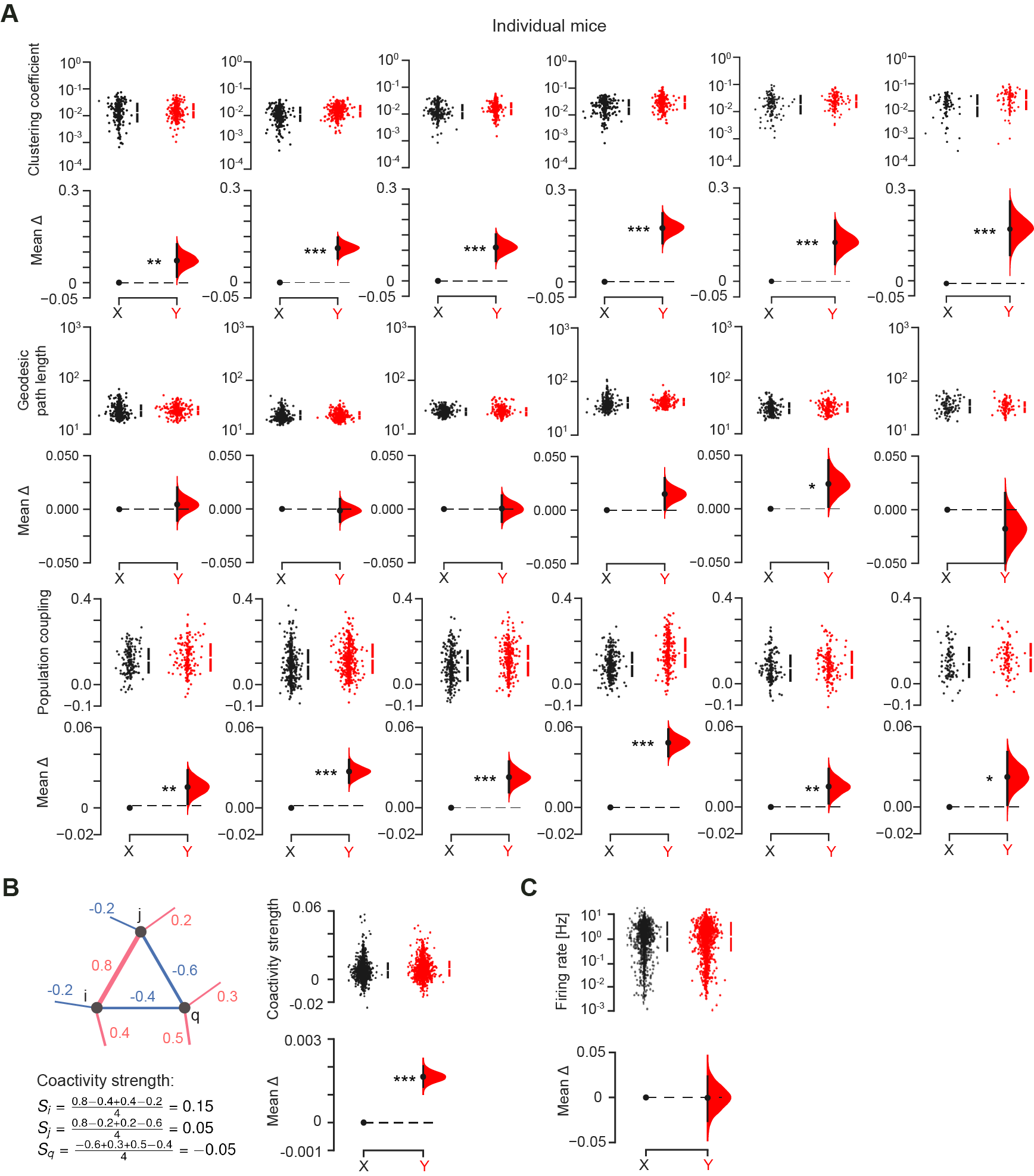


**Fig. S5. Animal-wise neuronal coactivity and population coupling were higher in context *Y*.**

**(A)** Estimation plots showing the effect size for the differences in mean clustering coefficient (first row), geodesic path length (second row), and population coupling (third row) between the neuronal coactivity graphs representing context *X* and those representing context *Y* across individual animals (one mouse per column).

**(B)** Coactivity strength analysis. *Left*: for clarity, an example subset of a coactivity graph is shown (top) with three neurons $i,j,q$ (nodes) along with their edges (lines) and signed pairwise coactivity values (numbers); for each node, the average weight $S$ of its edges is calculated (bottom). *Right*: estimation plot showing the effect size for the difference in coactivity strength levels in context *Y* compared to context *X*.

**(C)** The higher mean pairwise coactivity strength (B) occurred while the mean firing rate of CA1 principal cells was not different across contexts.

*** P<0.001, two-sided paired permutation test, 6 mice, 2506 CA1 principal cells.


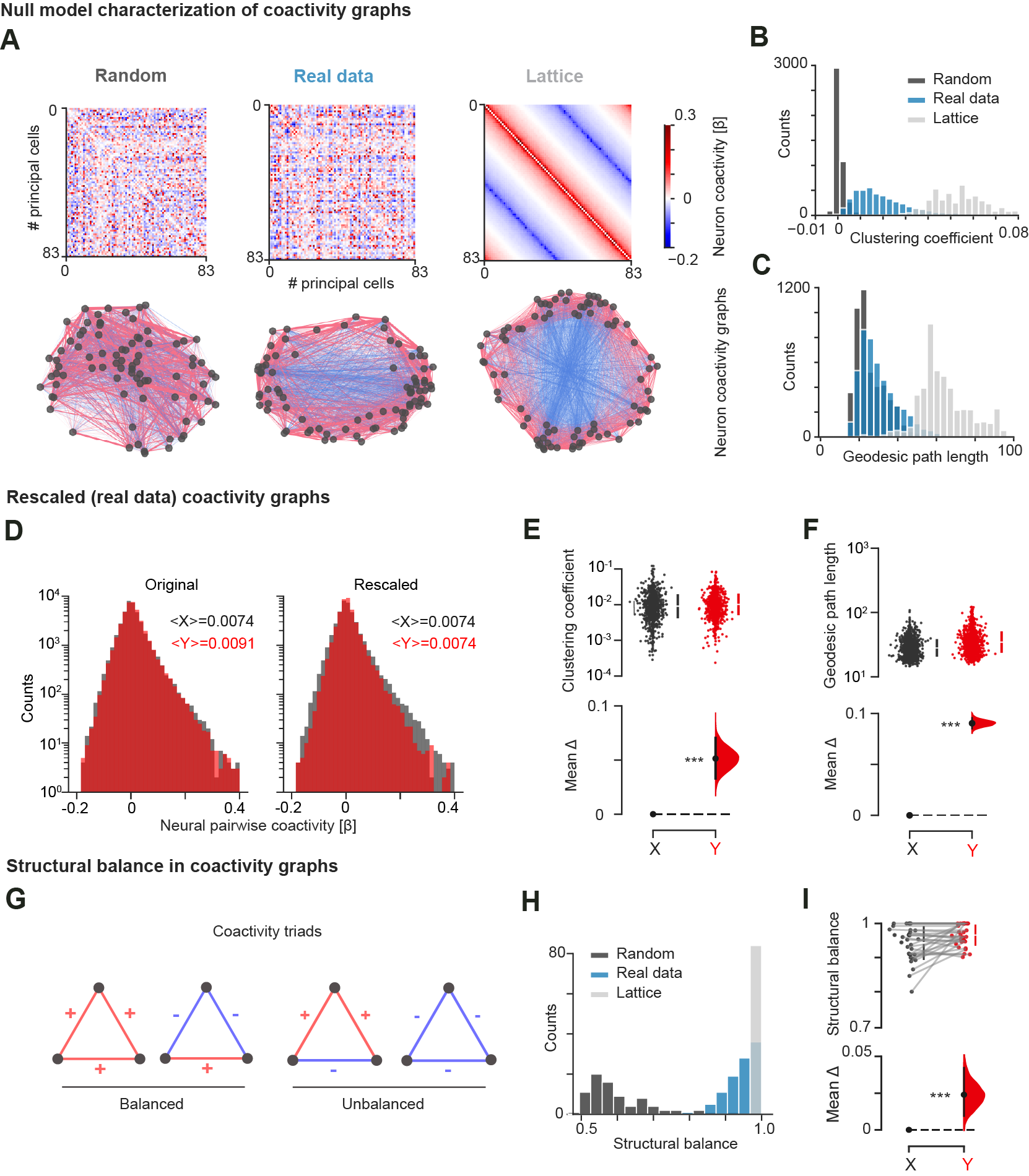


**Fig. S6. The topology of hippocampal coactivity in context *Y* converged towards that of a regular lattice.**

We conducted three additional analyses to further test the notion that the topology of hippocampal coactivity graphs developed in context *Y* with Hfd conditioning progressed towards the structure of a regular lattice.

**(A-C) Null models (random and lattice equivalent graphs) characterization.** We compared the real data networks obtained from our recordings to both their lattice and their random equivalents (see Methods). For one example session in context *Y* (**A**) are shown the principal cell coactivity matrices (top) and the corresponding graphs (bottom) for the random equivalent (left column), the real data (middle column; another example day of real data graph is shown in Fig. 2E), and the lattice equivalent (right column) of the CA1 network recorded that day. The distribution of clustering coefficients (**B**) and geodesic path lengths (**C**) are shown for the real data graphs obtained for all contexts, along with their random shuffled and lattice equivalents. Note that real data networks are overall featured by clustering coefficients closer to those of lattice networks while their geodesic path lengths resembled those of random networks, thus indicating a small-world topology (*16*, *18*).

**(D-F) Rescaled (real data) coactivity graphs.** We investigated whether the lattice-like topology of the neuronal coactivity structure observed in context *Y* (Fig. 2H, I) is merely explained by an increase in the coactivity values (Fig. S5B). To assess this, we analytically rescaled the edges of *Y* graphs to match the coactivity values of *X* graphs (**D**). With this*,* neuronal graphs in *Y* continued to display a higher clustering coefficient (**E**) but also a longer geodesic path length (**F**). Notably, this result suggested that the topological changes developed in context *Y* with Hfd conditioning (i.e., higher clustering coefficient and no significant change in geodesic path length; Fig. 2H, I) are not mere consequences of increased pairwise coactivity in the hippocampal network*.*

**(G-I) Structural balance in coactivity graphs.** Hippocampal coactivity graphs are composed of both correlated and anti-correlated spike trains (i.e., positive and negative edges). We thus further evaluated the structural balance (*67*) of these (signed) graphs, thresholding them to keep the top 10% of the highest magnitude links (both positive and negative) to then quantify the proportion of balanced triads (with an even number of negative edges) (**G**). As expected, the lattice equivalent graphs were fully balanced while the random ones were the most unbalanced; we observed that the real data graphs lied in between, closer to the lattice ones **(H).** Furthermore, real data coactivity graphs of context *Y* displayed a higher structural balance than those of context *X* **(I)**, supporting the idea that the hippocampus network shifted towards a more rigid, lattice-like topology in context *Y*.

***P<0.001, two-sided paired permutation test, 6 mice, 2506 CA1 principal cells.


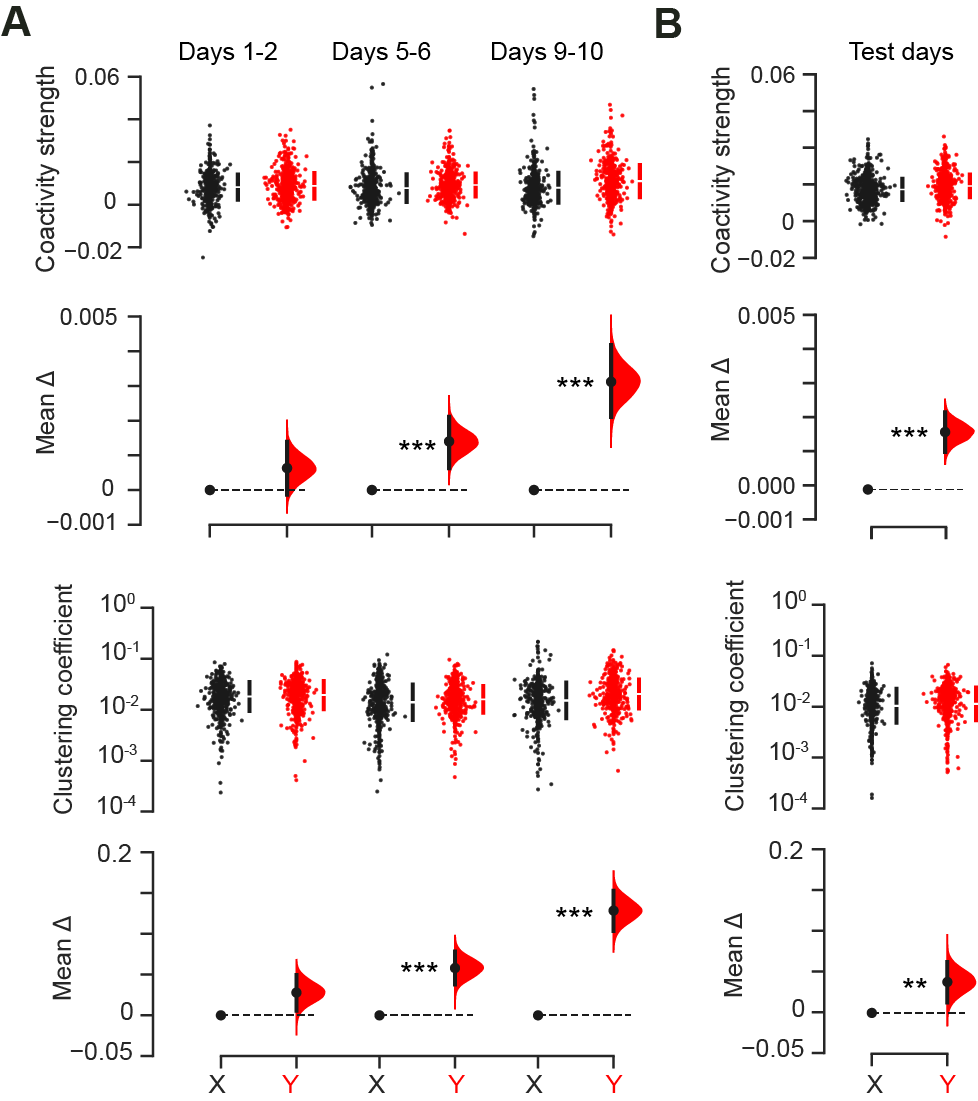


**Fig. S7. The topological changes affecting coactivity graphs develop during conditioning.**

We observed that the mean clustering coefficient (top) and the individual neuron coactivity strength (bottom) increased in context *Y* compared to *X* over the conditioning days (A) to continue affecting neuronal graphs during the re-exposure session in post-conditioning test days (B).

***P<0.001, **P<0.01, two-sided paired permutation test, 2506 CA1 principal cells from 6 mice.


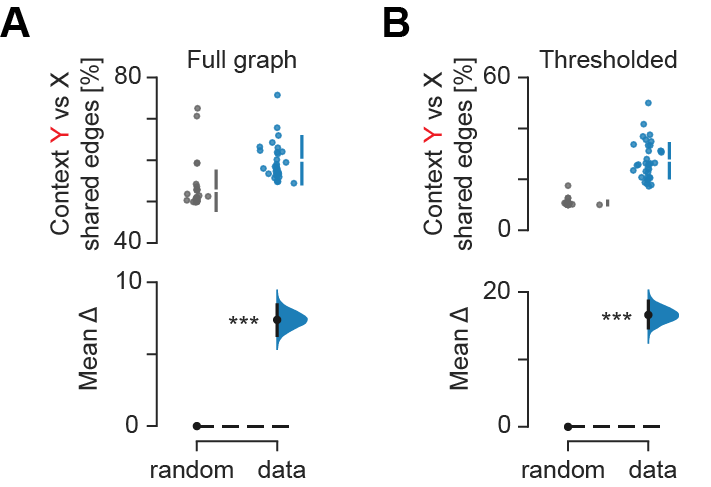


**Fig. S8. Neuronal graphs share some common coactivity structure across contexts.**

We further explored the relation between the population correlation structure underlying the neuronal graphs of context *X* versus those of context *Y*. Shown are the proportion of shared coactivity edges (i.e., common node-to-node same-sign edges) between the original (full dense) graphs of both context *X* and *Y* (**A**); and the proportion of these shared edges that remain in the corresponding binarized graphs (**B**; after thresholding the graphs to maintain only the 10% most positive links). For both analyses, we compared the results from the real data (population recorded in both context *X* and *Y*) graphs with their control graphs obtained by randomizing node-to-node edges in the original graphs. With both approaches, real data graphs for context *X* and *Y* (“data”) contained a higher proportion of shared structure than their random counterparts do (“random”). This shared structure might represent a backbone of correlated activity expressed across contexts. ***P<0.001, two-sided paired permutation test, 6 mice.


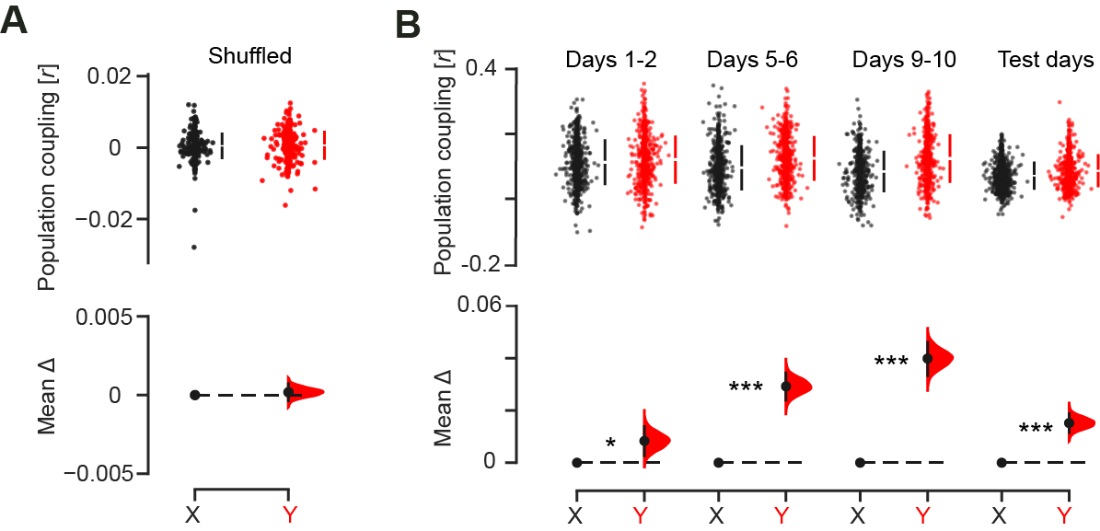


**Fig. S9. Population coupling of individual neurons increases during the 10-day conditioning and is maintained thereafter.**

**(A)** Shuffling the spike times across neurons and theta cycles, while preserving each neuron’s mean rate and the population rate distribution, cancelled the increased average population coupling of individual neurons seen in context *Y* (Fig. 2J). For this control analysis, the activity of each neuron was circularly shuffled independently with a delay drawn from a uniform distribution between 10 and 100 theta cycles. This result indicates that the increased population coupling seen in context *Y* compared to *X* (Fig. 2J) reflected stronger cross-neuron spiking relationship.

**(B)** Heightened population coupling developed across conditioning days to continue marking the re-exposure to context *Y* before any test during post-conditioning days. *P<0.05,

***P<0.001, two-sided paired permutation test, 2506 CA1 principal cells from 6 mice.


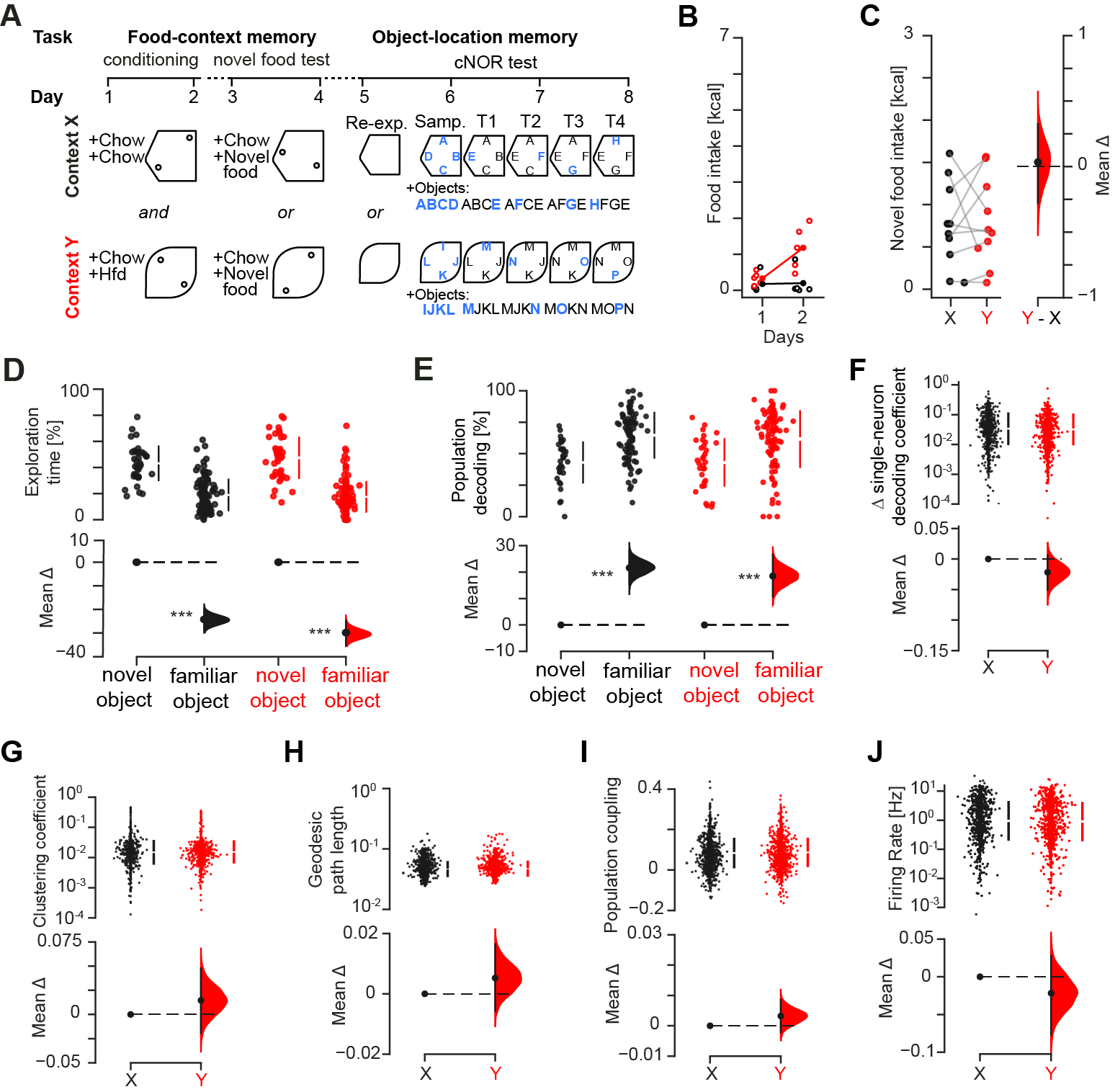


**Fig. S10. A 2-day food-context conditioning did not affect novel object preference and CA1 population activity.**

(**A**) Layout of the experimental paradigm (as in Fig. 1B and fig. S2A) but using only 2 days of food-context conditioning in five additional mice.

**(B)** Corresponding food intake during conditioning (each data point represents one mouse).

(**C)** Estimation plot showing the effect size for the difference in novel food intake across context *X* and *Y* after 2-day conditioning. Mice did not show feeding bias in context *Y* over *X*.

(**D**) Percentage of exploration time with novel and familiar objects during the subsequent cNOR tests in context *X* (black) versus context *Y* (red). Mice exhibited successful novel object preference in both contexts.

(**E**) Population classification accuracy of object-location compound in test $n$ by GLM trained in session $n-1$ (as in Fig. 1G; each data point represents one object). The object-location decoding using the spiking activity of CA1 principal cells was similar across the two contexts.

**(F)** The magnitude of single-neuron contribution to the population object-location classification was similar in both contexts. This contrasted with the difference obtained after 10 days of conditioning (Fig. 1J).

**(G, H)** The population coactivity structure remained similar in context *Y* versus *X* following the 2-day conditioning, as reported by the similar clustering coefficient of the corresponding neuronal graphs (G). The geodesic path length was also similar across contexts (H).

**(I, J)** Single-neuron coupling to population (I) and firing rate (J) also remained similar across the two contexts. ***P<0.001, two-sided paired permutation test, 5 mice, 1389 CA1 principal cells.


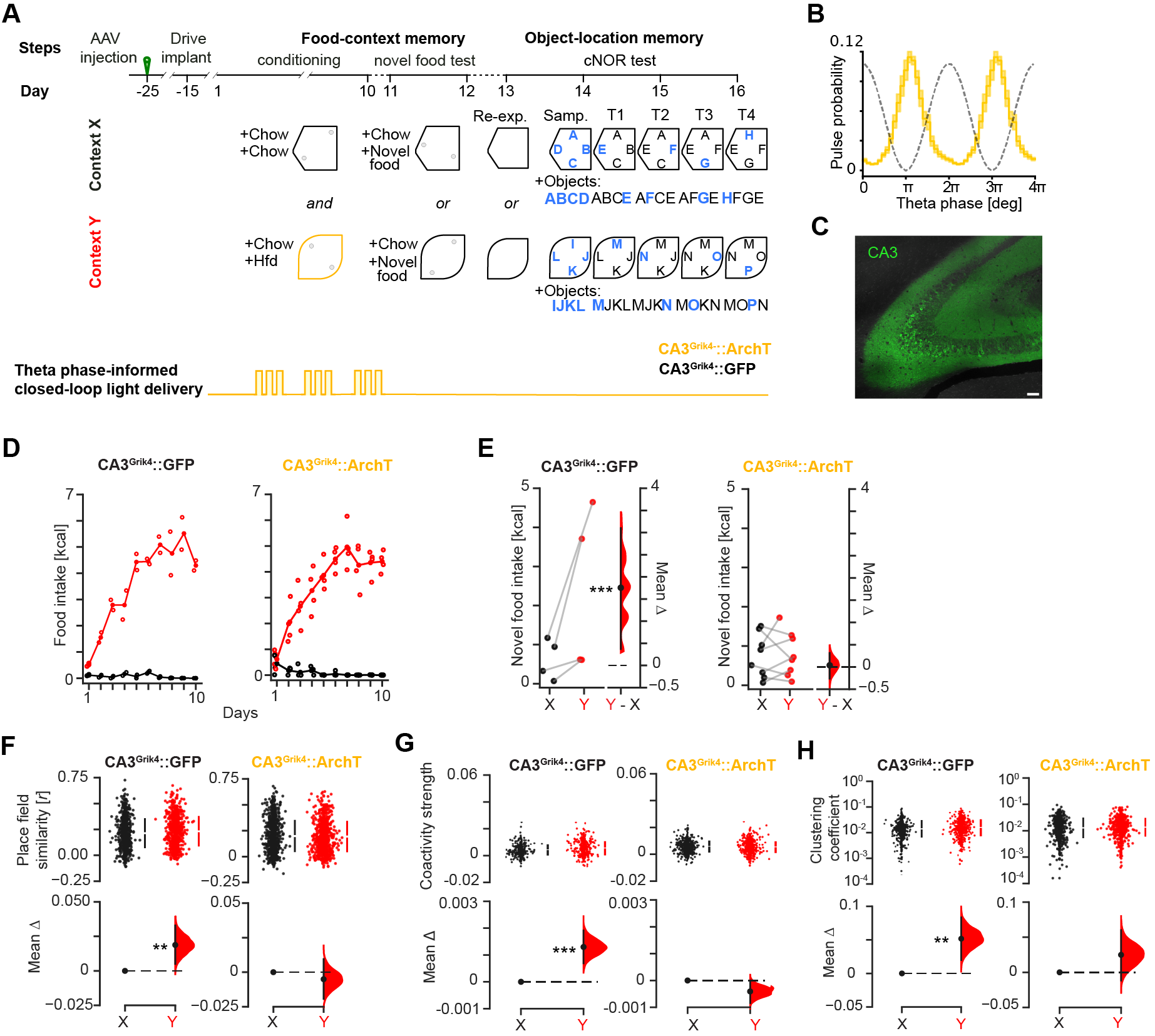


**Fig. S11. Contribution of CA3 during conditioning to subsequent CA1 coactivity.**

(**A**) Experimental layout. We used Grik4-Cre transgenic mice to target CA3 with either the ArchT-GFP construct or its GFP-only control; subsequent implantation of a 14-tetrode microdrive including two optic fibers allowed monitoring CA1 neuronal ensembles with theta phase-informed bilateral light delivery to CA3.

**(B)** Light pulse midpoint probability as a function of ongoing theta phase for closed-loop CA3 light delivery during each Hfd conditioning session in context *Y*.

(**C**) Low magnification confocal image showing CA3 principal cells transduced with ArchT-GFP (green). One optical section; scale bar, 100 μm.

(**D**) Food intake over the 10-day conditioning in context *X* (black) versus context *Y* (red) for the CA3^Grik4^::GFP control mice (left) and the CA3^Grik4^::ArchT mice (right). Each data point represents one mouse.

(**E)** Estimation plots showing the effect size for the difference in novel food intake across context *X* and *Y* in the post-conditioning novel food test for the CA3^Grik4^::GFP control mice (left) and the CA3^Grik4^::ArchT mice (right). Each data point represents one mouse.

**(F)** Place field similarity between the place maps expressed by individual CA1 principal cells in each pair of contiguous cNOR sessions in context *X* versus context *Y* (each data point represents one cell; as in Fig. 2B). This PFS analysis indicates that applying theta phase-informed closed-loop suppression of CA3 principal cells during each Hfd conditioning session subsequently restored in CA3^Grik4^::ArchT mice (right), but not CA3^Grik4^::GFP mice (left), similar levels of spatial remapping across cNOR sessions in context *Y* compared to *X*.

**(G)** In CA3^Grik4^::ArchT mice, we observed that following CA3 suppression throughout conditioning days, the CA1 coactivity strength in context *Y* was similar to that in context *X* during post-conditioning test days. This was not the case in CA3^Grik4^::GFP mice, which showed higher CA1 coactivity strength in context *Y* compared to *X*.

**(H)** Likewise, we observed that this intervention leveled the CA1 mean clustering coefficient across the two contexts in CA3^Grik4^::ArchT mice (right), but not in CA3^Grik4^::GFP mice (left), during subsequent post-conditioning test days.

***P<0.001, two-sided paired permutation test, 1548 CA1 principal cells from 4 CA3^Grik4^::ArchT mice versus 881 CA1 principal cells from 2 CA3^Grik4^::GFP mice.


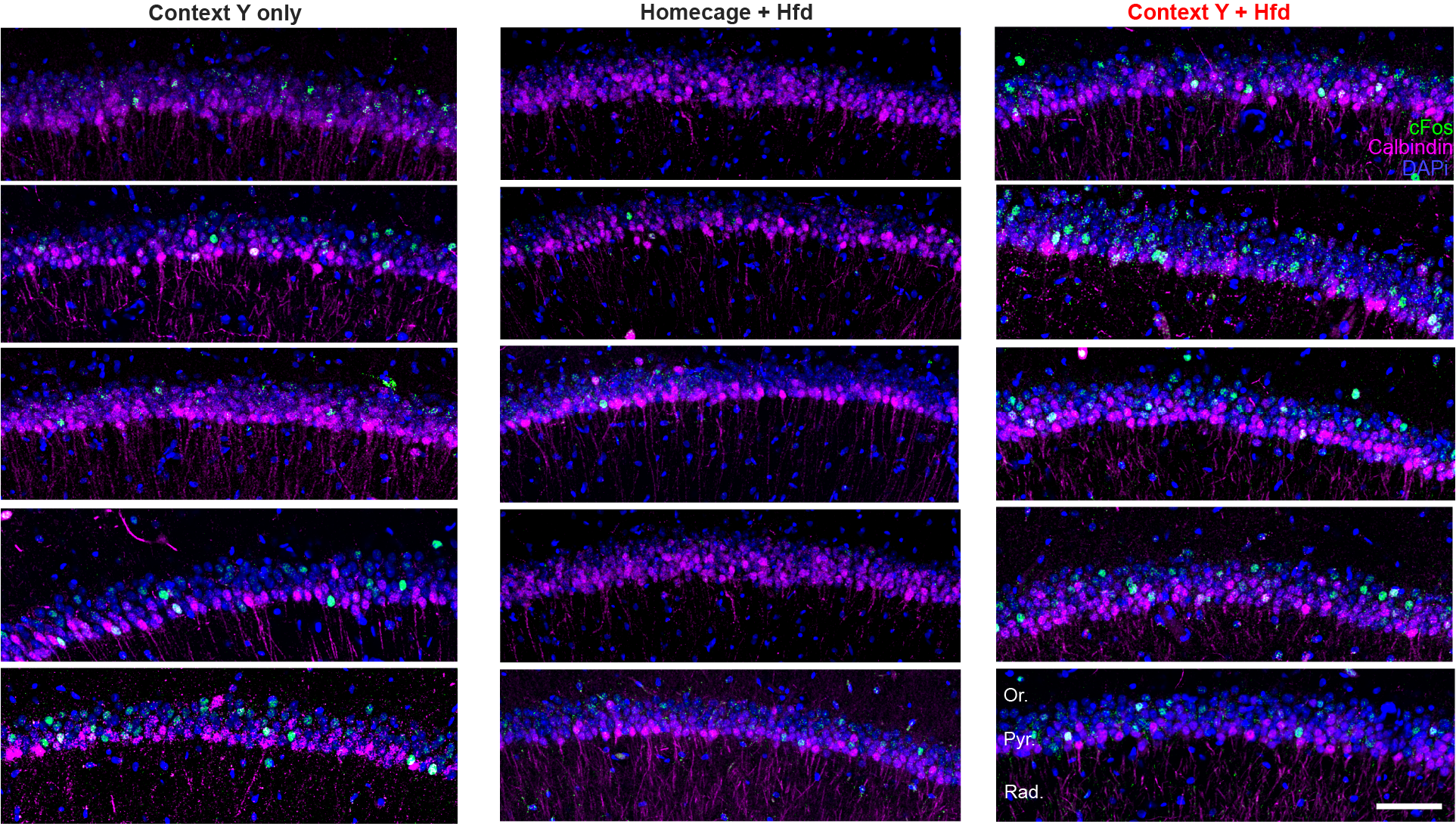


**Fig. S12. Endogenous cFos expression in the CA1 *pyramidale* layer with respect to context *Y* and Hfd.**

Confocal pictures showing cFos–expressing neurons in the CA1 *pyramidale* layer for fifteen additional mice (one picture per mouse) after either 10-day exploration of context *Y* without Hfd (left), 10-day consumption of Hfd in the homecage (middle), or 10-day exploration of context *Y* with Hfd (right). Each mouse session day was 30 minutes. Cell nuclei stained with DAPI (blue). Scale bar: 50 μm. CA1 layers: *Oriens*, Or.; *Pyramidale*, Pyr.; *Radiatum,* Rad.


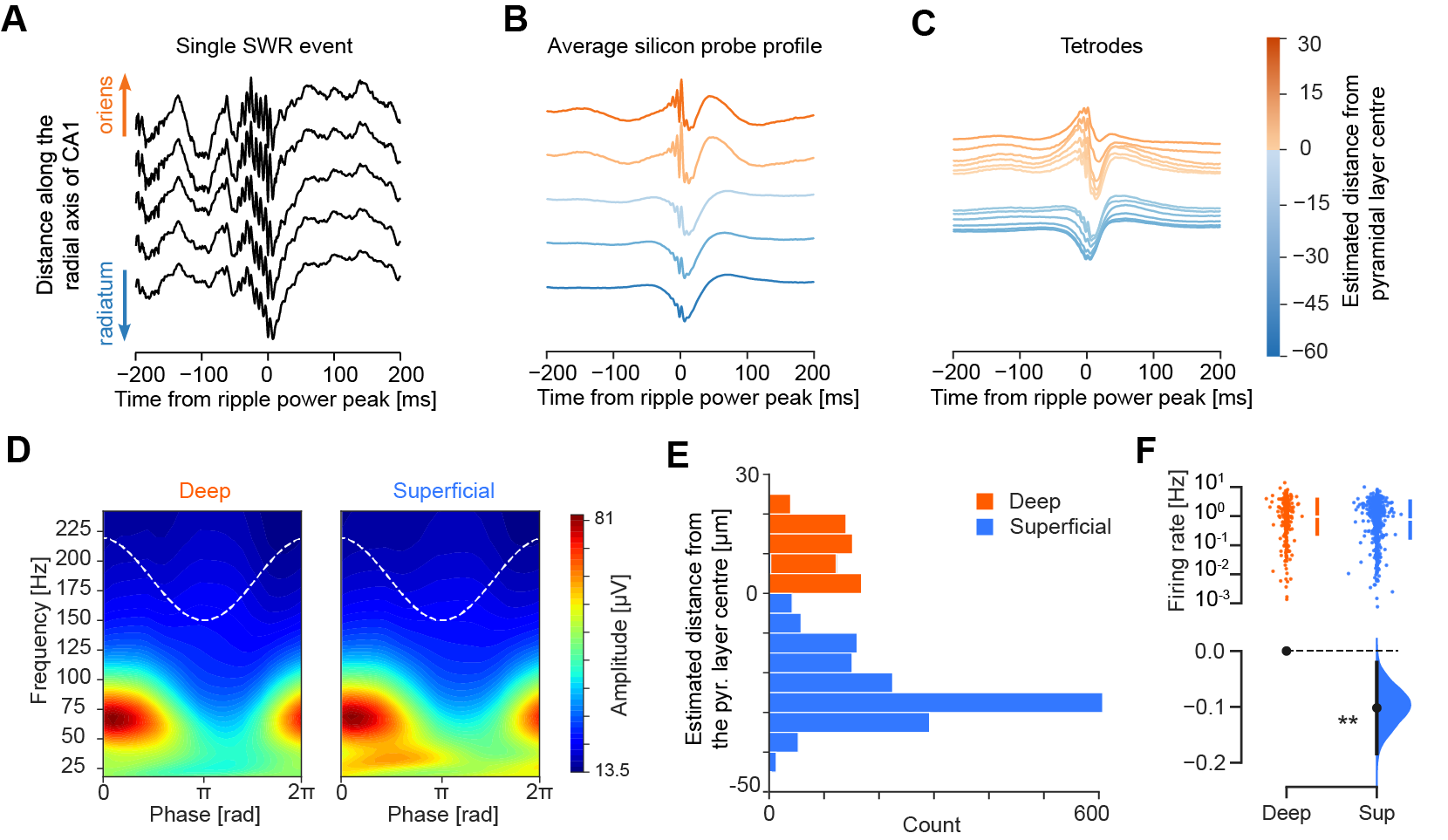


**Fig. S13. Identification of deep versus superficial CA1 *pyramidale* sublayer cells.**

**(A-C)** We estimated the position (depth) of individual tetrode-recorded principal cell soma by leveraging silicon probe recordings with known spacing between the recording sites along a linear shank (*55*). From these silicon probe recordings (n=6 mice), we first acquired the laminar profile of both sharp-wave ripples (SWRs) and theta oscillations detected in the LFPs. Using these average LFP waveforms, we then established a feature trajectory along the radial axis of the CA1 hippocampus (*55*). Within the CA1 pyramidal layer, the information about depth primarily relies on the laminar profile of the SWR waveforms (A, B). Single tetrodes (and thus their recorded neurons) are then projected onto this trajectory to estimate their depth (C).

**(D)** We further computed the average spectral profile for deep and superficial *pyramidale* tetrodes. This independent analysis showed that the spectral content of the LFP signals recorded from deep and superficial tetrodes differed with respect to the presence of slow gamma oscillations.

**(E)** Corresponding distribution of CA1 principal cells with respect to the estimated depth of their recording tetrodes.

**(F)** CA1 principal cells recorded from the superficial *pyramidale* sublayer (closer to *radiatum*) showed lower firing rates (measured during theta oscillations of active exploration) compared to their deep sublayer counterparts (closer to *oriens*), in line with previous work (*15*, *28*, *73*, *74*). **P<0.01, two-sided paired permutation test. N = 635 deep sublayer cells and 1,871 superficial sublayer cells from 6 mice.


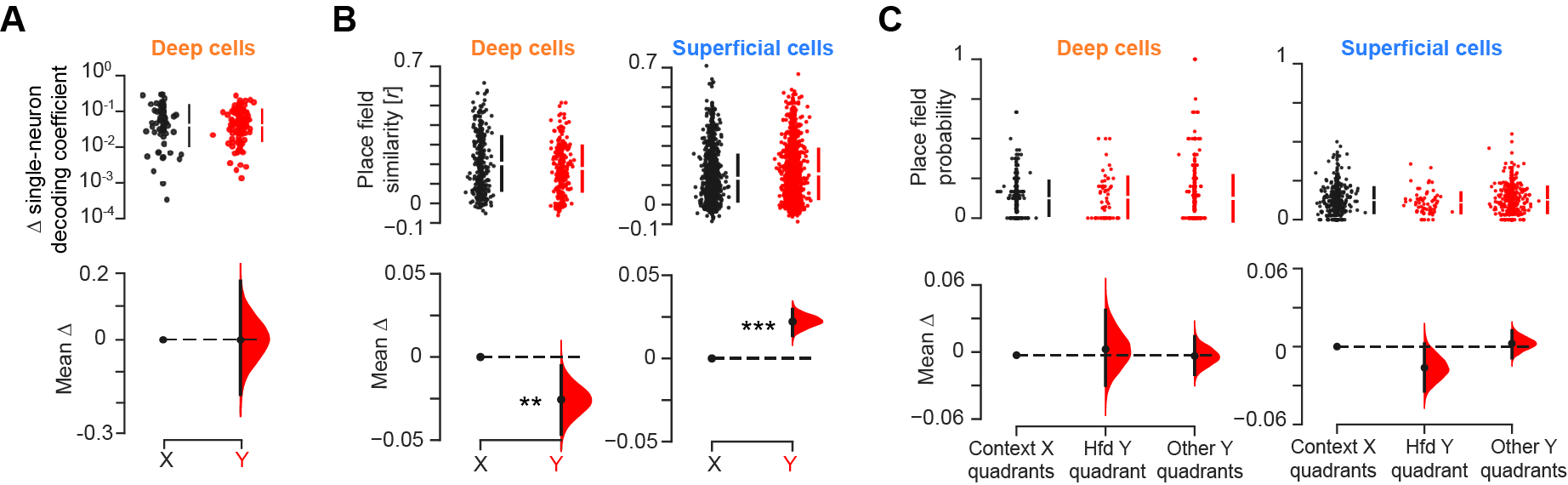


**Fig. S14. Object-location decoding contribution of deep *pyramidale* sublayer cells and place map stability across cNOR sessions for the two CA1 principal cell subpopulations.**

**(A)** Estimation plot showing the change in the magnitude of single-neuron classification contribution to novel object-location population decoding for CA1 deep *pyramidale* sublayer cells, quantified as the difference in their GLM coefficients in either context *X* or Y (see Fig. 4C for the superficial cells).

**(B)** Likewise, shown for both context *X* and *Y* is the place field similarity of the spatial maps expressed by principal cells in the deep versus the superficial sublayers was assessed across cNOR sessions in either context *X* or context *Y* (as in Fig. 2B) following the 10-day conditioning. This shows that the greater stability of the CA1 place maps across cNOR sessions in context *Y* (Fig. 2B) is strongly explained by the higher place field similarity (i.e., lower cross-session remapping) of superficial cells.

**(C)** The probability for deep and superficial cell to represent the previously experienced Hfd location while performing cNOR tests in context *Y* (“Hfd *Y* quadrant”) was not significantly different from the probability to represent any other locations in context *Y* (“Other *Y* quadrants”) or in context *X* (“Context *X* quadrants”). Same place field analysis as in fig. S4B.

***P<0.001, **P<0.01, two-sided paired permutation test. N = 635 deep sublayer cells and 1,871 superficial sublayer cells from 6 mice.


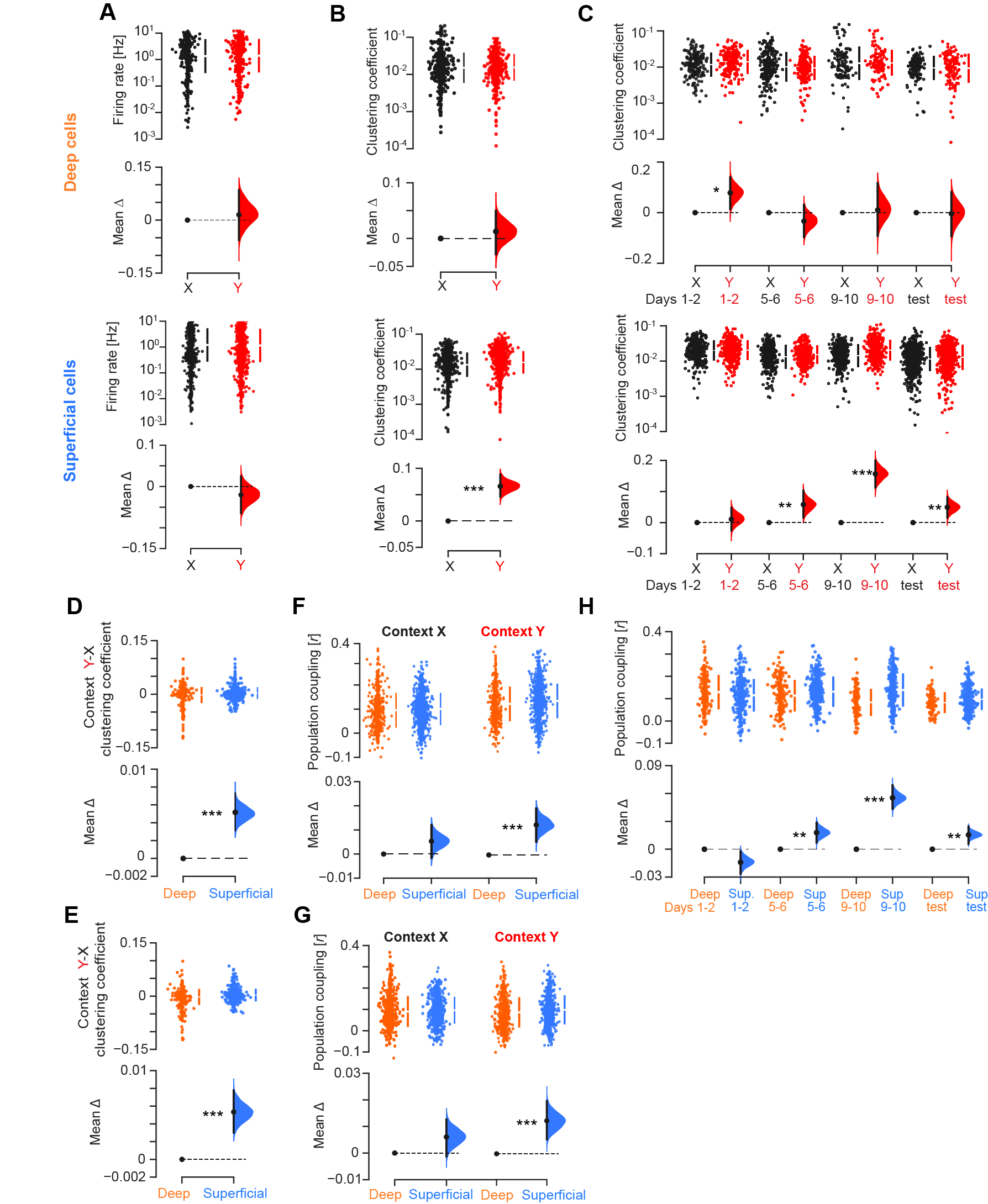


**Fig. S15. Comparison of deep versus superficial CA1 cell activity.**

**(A)** Estimation plots showing no significant change in the firing rate of principal cells in the deep (top) and the superficial (bottom) CA1 *pyramidale* sublayers between the Hfd-paired context *Y* compared to the chow-paired context *X*.

**(B)** Likewise, shown is the coactivity clustering coefficient for deep (top) and superficial (bottom) *pyramidale* sublayer cells between context *X* and *Y*.

**(C)** The clustering coefficient for deep cells (top) tended to be similar between context *X* and *Y* from conditioning to testing, albeit higher in *Y* during the first two days of conditioning. The coactivity clustering coefficient of superficial cells (bottom) increased in context *Y* compared to *X* across task days.

**(D)** Change in clustering coefficient for deep versus superficial cells in context *Y* minus that in context *X*. All cells included.

**(E)** Same as (D), but to control for the different numbers of deep versus superficial cells (fig. S13F), we resampled these two populations to match their sample size in a day-by-day fashion (N = 635 cells for each population). This confirmed that superficial cells display a higher clustering coefficient compared to deep pyramidale sublayer cells.

**(F)** Likewise, shown are the change in population coupling for deep versus superficial cells in context *X* and *Y*. All cells included.

**(G)** Same as (F), but to control for the different numbers of deep versus superficial cells, we also resampled these two populations to match their sample size in a day-by-day fashion (N = 635 cells for each population). This confirmed that superficial cells display a higher population coupling compared to deep pyramidale sublayer cells.

**(H)** Population coupling of superficial cells increased compared to that of deep cells across conditioning and test days in context *Y*.

***P<0.001, **P<0.01, *P<0.05, two-sided paired permutation test. N = 635 deep sublayer cells and 1,871 superficial sublayer cells from 6 mice.


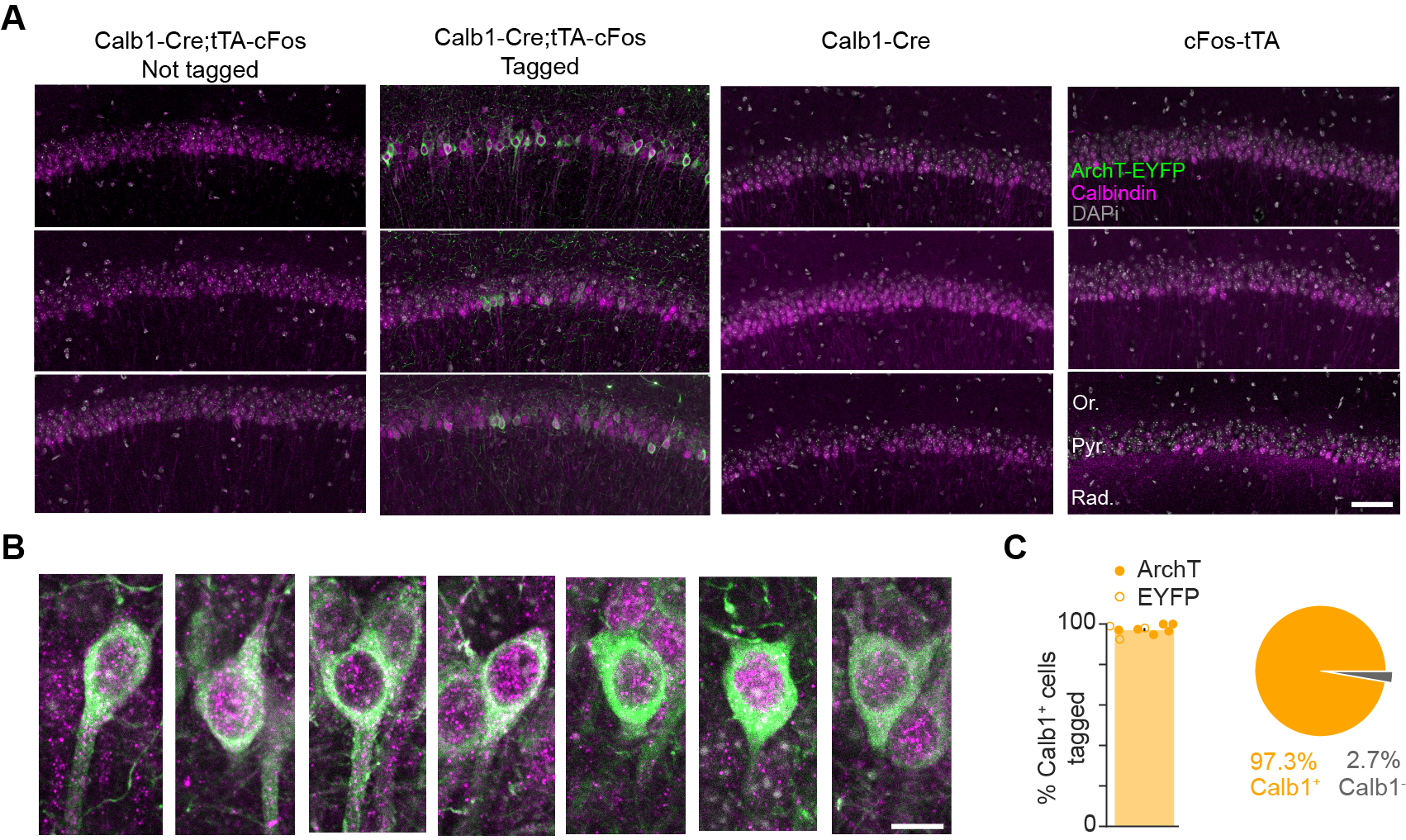


**Fig. S16. Histological validation of the intersectional optogenetic strategy used to tag CA1 superficial *pyramidale* sublayer cells recruited during Hfd-context *Y* conditioning.**

(**A, B**) To validate the intersectional optogenetic targeting of CA1 superficial pyramidale sublayer cells during Hfd-context Y conditioning (Fig. 5), we injected the hSyn-Con/Fon-ArchT-EYFP and associated TRE3G-FlpO constructs in four groups of mice (from left to right): (1) the double-transgenic Calb1-Cre;cFos-tTA mice exposed to Context *Y* with Hfd while always on homecage Dox diet (i.e., “not tagged”); (2) the double-transgenic Calb1-Cre;cFos-tTA mice exposed to Context *Y* with Hfd while transiently not on homecage Dox diet (i.e., “tagged”); (3) the mono-transgenic Calb1-Cre mice and (4) the cFos-tTA mice, also both exposed to Context *Y* with Hfd. Shown in (A) are low magnification confocal pictures for ArchT-EYFP expression (green) and Calbindin immunostaining (magenta) in the CA1 *pyramidale* layer for twelve mice (one picture per mouse; each column represents one group) one week after 30-min exposure to context *Y* with Hfd. ArchT-EYFP was selectively expressed in the pyramidale layer of the Context *Y* “tagged” Calb1-Cre;cFos-tTA mice but not the other three groups. Shown in (B) are high magnification confocal pictures for representative examples of CA1 cells expressing ArchT-EYFP (green) and immuno-positive for Calbindin (magenta) from the double-transgenic Calb1-Cre;cFos-tTA mice tagged in Context *Y* with Hfd (second column from the left in A). Scale bar: 50 μm (A) and 10 μm (B). Cell nuclei stained with DAPI (gray). CA1 layers: *Oriens*, Or.; *Pyramidale*, Pyr.; *Radiatum,* Rad.

(**C**) In Context *Y*-tagged Calb1-Cre;cFos-tTA mice, 97.26±0.77% of the tagged CA1 *pyramidale* layer cells (with ArchT-EYFP in 6 mice, or EYFP-only in 3 mice) were immuno-stained with the Calb1 antibody (with 90.98±0.93% of these cells located in the superficial sublayer and 9.02±0.93% in the deep sublayer).


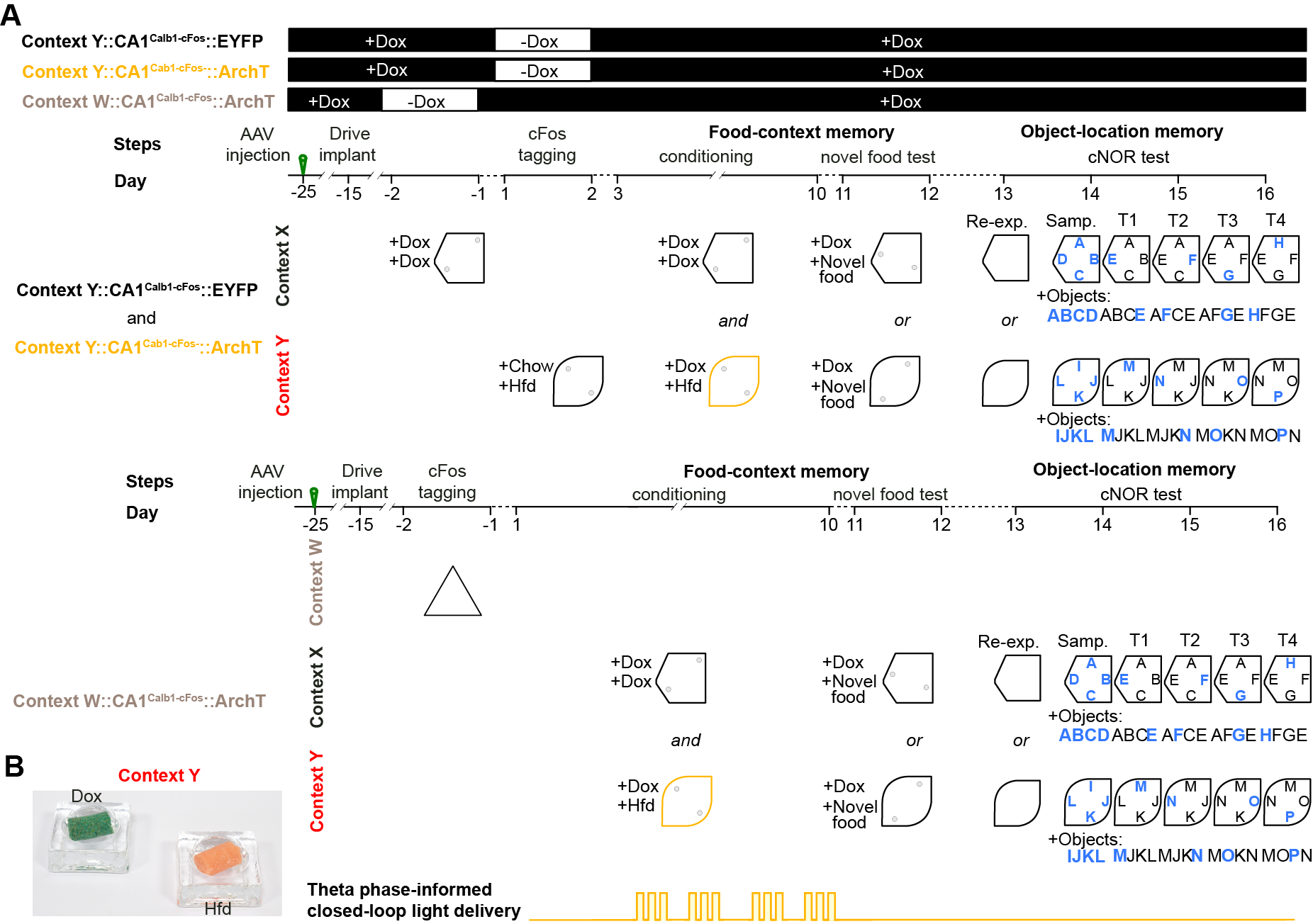


**Fig. S17. Experimental framework for theta phase-informed optogenetic feedback in CA1^Calb1-cFos^ mice.**

(**A**) Experimental layout. We used double-transgenic Calb1-Cre;cFos-tTA mice fed with doxycycline (Dox) containing food pellets in their homecage. Using an intersectional targeting strategy (Fig. 5A), we targeted the CA1 of these mice with either the ArchT-EYFP construct or its EYFP-only control; subsequent implantation of a 14-tetrode microdrive including two optic fibers allowed monitoring CA1 neuronal ensemble with theta phase-informed bilateral light delivery. To tag CA1 neurons in the Hfd-paired context *Y* with ArchT-EYFP (in Context*Y*::CA1^Calb1-cFos^::ArchT mice) or EYFP-only (in Context*Y*::CA1^Calb1-cFos^::EYFP mice), the homecage feeding with Dox pellets was replaced with regular Chow pellets during the 48-hour period corresponding to days 1 and 2 of Hfd-context *Y* conditioning. To restrict this tagging to context *Y*, mice were not exposed to the other contexts during this off-Dox period. For this reason, the context *Y* tagged mice started their day 1 and 2 conditioning of Chow in context *X* in the two days before, while still on Dox diet (i.e., they were not exposed to context *X* for the two days corresponding to Hfd-context *Y* tagging). CA1 light delivery was then actuated by real time tracking of theta phase while mice continued to explore context *Y* with Hfd from conditioning day 3 onward. In a third group of mice, we tag CA1 neurons recruited in a neutral (i.e., unrelated to the contextual feeding task) context *W* with ArchT-EYFP (Context*W*::CA1^Calb1-cFos^::ArchT mice). For this, the homecage feeding with Dox pellets was transiently replaced with regular Chow pellets for the 48-hour period corresponding to days -1 and -2 with respect to the Hfd-context *Y* conditioning. To restrict neuronal tagging to context *W*, these mice were not exposed to the other contexts during this off-Dox period. Context *W* tagged mice then started their conditioning in context *X* versus context *Y* in the following days while back on Dox diet, with CA1 light delivery actuated by real time tracking of theta phase while mice explored context *Y* with Hfd in each conditioning session.

**(B**) The two food containers used to provide mice with a choice between one Dox-diet pellet versus one Hfd pellet in context *Y*.


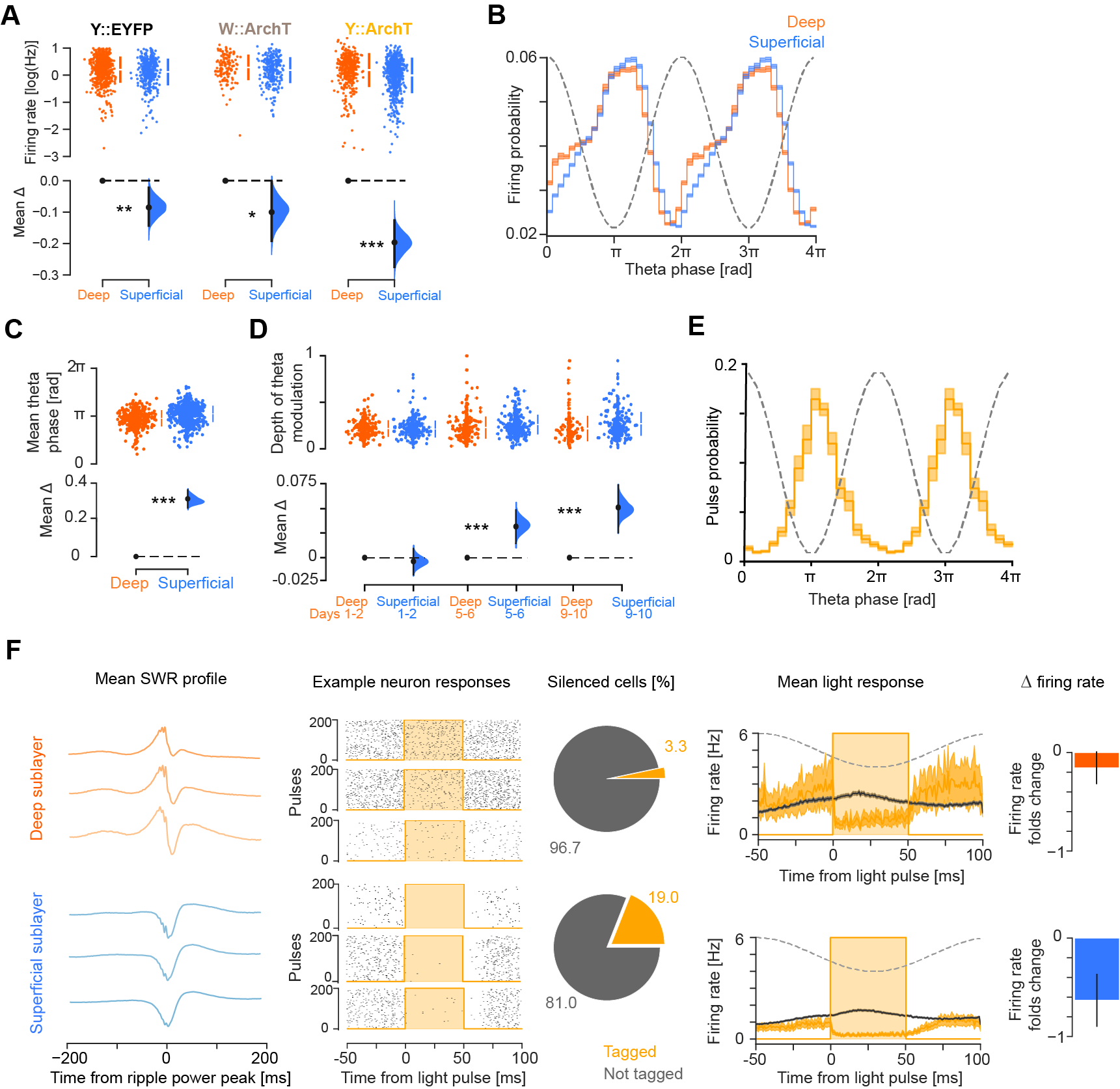


**Fig. S18. Firing activity of deep and superficial CA1 principal cells related to the theta phase-informed manipulation.**

(**A**) Estimation plot showing that the baseline firing rate of CA1 superficial cells (blue) is lower than CA1 deep cells (orange) in the three groups of CA1^Calb1-cFos^ mice engaged in the theta phase-informed optogenetic feedback framework (fig. S17). Baseline firing rate calculated in the absence of light delivery. This result replicates the difference observed in the initial cohort of mice (fig. S13F).

(**B-D**) Basic theta-paced firing activity of CA1 principal cells recorded in the original, non-optogenetically modulated mice. Firing probability of deep versus superficial CA1 cells as a function of theta phase (B) and mean theta phase coupling difference between these two neuronal populations (C). Note that superficial cells tend to fire slightly later in the theta cycle. The theta modulation of CA1 superficial cell spiking increases compared to CA1 deep cells as the Hfd conditioning progresses in context *Y* (D).

(**E**) Probability of real time light delivery (50-ms pulse midpoint) as a function of ongoing theta phase during Hfd conditioning in context *Y*.

**(F)** Effect of the closed-loop intervention on the activity of CA1 principal cells recorded in the deep versus superficial CA1 pyramidale sublayer (top and bottom rows, respectively) of the CA1^Calb1-cFos^ mice tagged with ArchT. From *Left* to *Right*: (i) Example SWR profiles used to locate individual tetrodes along the CA1 radial axis (*15*). (ii) Examples raster plots for principal cells recorded from deep versus superficial tetrodes and showing single-neuron firing responses to light delivery. (iii) Pie charts showing the percentage of ArchT-tagged principal cells (i.e., cells silenced by light delivery) per CA1 sublayer; note that the few silenced cells recorded in the deep sublayer are likely to be Calbindin-1 cells (fig. S16C). (iv) With respect to the onset of light pulse delivery, shown is the time course of the average firing activity of tagged (yellow) and non-tagged (gray) principal cells; for clarity, cosines (dashed) indicate theta phase reference using one cycle (note the theta modulation of the firing rate). (v) Corresponding folds change in firing rate for the deep versus superficial cell subpopulations.

***P<0.001, **P<0.01, *P<0.05, two-sided paired permutation test; n = 1097 CA1 principal cells (413 deep with 684 superficial) from 5 Context*Y*::CA1^Calb1-cFos^::ArchT mice (Y::ArchT), 1007 (416 deep with 591 superficial) from 4 Context*Y*::CA1^Calb1-cFos^::EYFP mice (Y::EYFP), 683 (231 deep with 452 superficial) from 3 Context*W*::CA1^Calb1-cFos^::ArchT mice (W::ArchT) (A, F); and 635 deep sublayer cells with 1,871 superficial sublayer cells from 6 mice (B-D).


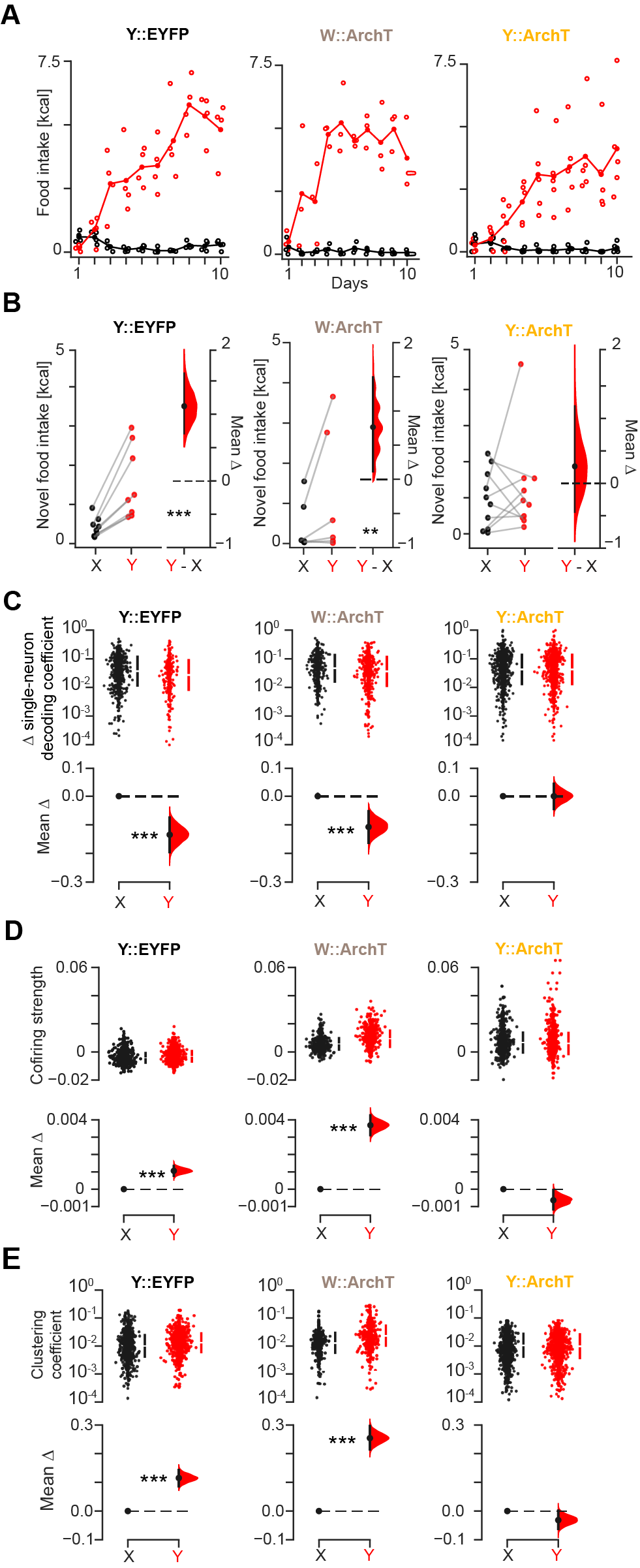


**Fig. S19. Optogenetic silencing of Context *Y*-tagged CA1 superficial cells during Hfd conditioning subsequently restored functional population activity.**

(**A**) Food intake over the 10-day conditioning in context *X* (black) versus context *Y* (red) for the Context*Y*::CA1^Calb1-cFos^::EYFP control mice (left), the Context*W*::CA1^Calb1-cFos^::ArchT mice (middle; mice tagged in context *W* with ArchT), and the Context*Y*::CA1^Calb1-cFos^::ArchT mice (right). Each data point represents one mouse.

(**B)** Estimation plots showing the effect size for the difference in novel food intake between context *X* and *Y* during the post-conditioning novel food tests for the three CA1^Calb1-cFos^ mouse groups. Each data point represents one mouse.

**(C-E)** Estimation plots showing (C) the change (update) in the magnitude of single neuron contribution to population object-location classification, quantified as the difference of the GLM coefficients in either context (see also Fig. 1J and methods), (D) coactivity strength, and (E) clustering coefficient. Interestingly, we noted that silencing in context *Y* the set of CA1 neurons tagged with ArchT in context *W* seemed to promote further population activity in context *Y* (compared to EYFP-only controls), in line with previous observation that manipulating a context-dependent set of principal cells impacts on neighboring sets of principal cells (*54*). For each estimation plot: *Upper*, raw data (*points*) with mean±SD (*vertical lines*); *Lower*, mean difference (*black-dot*) with 95% CI (*black-ticks*) and bootstrapped sampling-error distribution (*filled-curve*) with respect to the (left-most) group-reference (*horizontal dashed line*; see Methods). ***P<0.001; two-sided paired permutation test; n = 1097 CA1 principal cells from 5 Context*Y*::CA1^Calb1-cFos^::ArchT mice, 1007 from 4 Context*Y*::CA1^Calb1-cFos^::EYFP mice, and 683 from 3 Context*W*::CA1^Calb1-cFos^::ArchT mice.


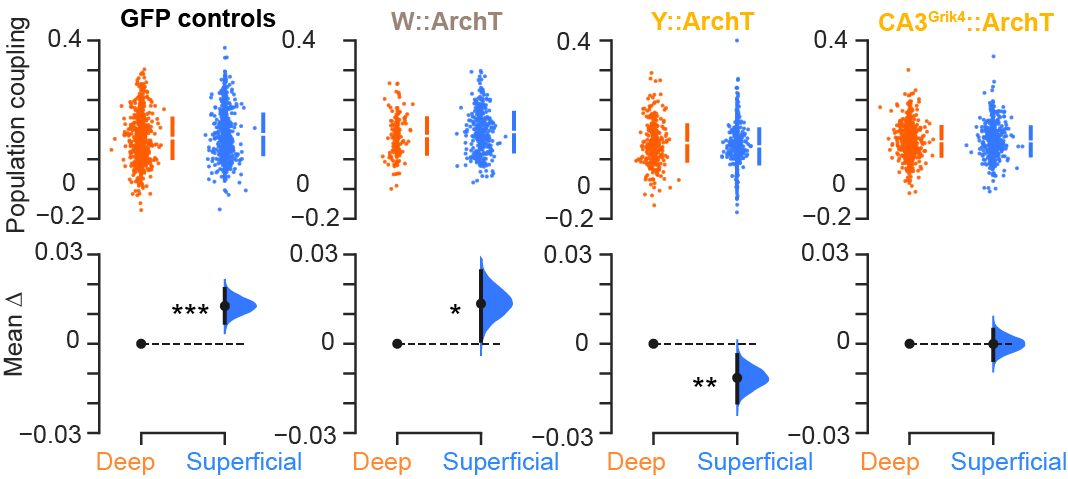


**Fig. S20. Population coupling of individual CA1 principal cells during post-conditioning test days.**

Following the 10-day conditioning marked by closed-loop light delivery in Hfd-context *Y* sessions, the population coupling of individual CA1 superficial cells remained stronger than that of CA1 deep cells in GFP control mice (Context*Y*::CA1^Calb1-cFos^::EYFP mice and CA3^Grik4^::GFP mice) as well as Context*W*::CA1^Calb1-cFos^::ArchT mice, which both showed impaired flexible object-location memory (Figs. 3D, 5E). This was no longer the case in Context*Y*::CA1^Calb1-cFos^::ArchT mice and CA3^Grik4^::ArchT mice, showing that rebalancing CA1 population coupling corresponded with restored memory flexibility.

***P<0.001, **P<0.01, *P<0.05, two-sided paired permutation test; n = 1097 CA1 principal cells (413 deep with 684 superficial) from 5 Context*Y*::CA1^Calb1-cFos^::ArchT mice (Y::ArchT), 1888 (811 deep with 1077 superficial) from 4 Context*Y*::CA1^Calb1-cFos^::EYFP mice and 2 CA3^Grik4^::GFP control mice (GFP controls), 683 (231 deep with 452 superficial) from 3 Context*W*::CA1^Calb1-cFos^::ArchT mice (W::ArchT), and 1548 (887 deep with 661 superficial) from 4 CA3^Grik4^::ArchT mice (CA3^Grik4^:ArchT).
